# Carboxylate positional isomerism in metallacycles governs hierarchical assembly pathways

**DOI:** 10.1039/d5sc05591a

**Published:** 2025-10-13

**Authors:** Lingran Liu, Meilin Yu, Wei Tuo, Yue Zhao, Fengmin Zhang, Yan Sun

**Affiliations:** a School of Nanoscience and Materials Engineering, Henan University Zhengzhou 450046 China elaine.sun@henu.edu.cn; b Department of Chemistry, University of Utah 315 South 1400 East, Room 2020 Salt Lake City Utah 84112 USA; c School of Chemistry and Chemical Engineering, Nanjing University Nanjing 210093 P. R. China; d Testing Center of Yangzhou University Yangzhou Jiangsu 225002 China

## Abstract

Positional isomerism plays a pivotal role in governing the assembly pathways of hierarchical architectures by modulating both the thermodynamic landscape and kinetic trajectories through precise spatial control of the constituent units, ultimately dictating the assembly efficacy and structural outcomes. Elucidating the underlying selectivity mechanisms of such isomerism offers fundamental insights into the rational design of advanced functional materials with tailored properties. Our findings reveal that isomeric variations in metal–organic cycles (MOCs) at the molecular scale trigger a cascade of structural effects—reconfiguring noncovalent interaction networks (particularly hydrogen bonding), diverting hierarchical assembly pathways, and ultimately generating distinct mesoscale architectures (extralong fibers and wide ribbons) with divergent physicochemical properties—thereby providing fundamental mechanistic insights into information transfer across length scales.

## Abstract 2

Positional isomerism plays a pivotal role in governing the assembly pathways of hierarchical architectures through precise spatial control of the constituent units, ultimately dictating the assembly efficacy and structural outcomes.

## Graphical abstract

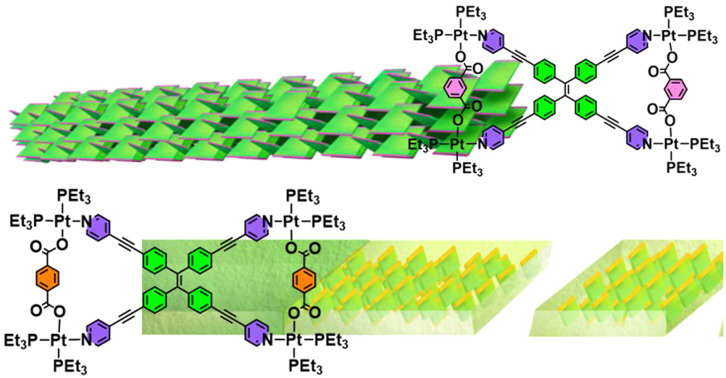

## Introduction

Positional isomers^1^ precisely capture the molecular details of signal transduction in organisms through allosteric regulation from the initial control of molecular conformation to hierarchical self-assembly that precisely regulates the construction of biological systems,^2^ ultimately achieving functional assembly at the organelle level. The three-tiered amplification mechanism of conformation–recognition–organization reveals the core regulatory principle of structure–dynamics–function, enabling the generation of exponential functional diversity from minute positional differences.^3^ In synthetic chemistry, positional isomers,^4^ which are molecular variants with subtle structural differences and distinct functionalities, reshape molecular design paradigms across multiple disciplines. Recent breakthroughs have demonstrated their extraordinary potential: in drug development, precisely engineered adrenaline positional isomers achieve subtype-selective GPCR activation;^5^ in energy science, the strategic arrangement of ammonium groups in perovskites has maximized the photovoltaic efficiency;^6^ and in nanomedicine, the self-assembly of porphyrin positional isomers enables spatiotemporally controlled combination therapies.^7^

To elucidate the mechanisms by which positional isomers function in hierarchical assembly, it is necessary to decipher the cascade transmission mechanisms underlying their dynamic assembly pathways. This process requires evaluating the amplification process from molecular conformational transitions to macroscopic functional assembly, in addition to mapping nonlinear configuration–function relationships at various assembly nodes, thereby achieving precise prediction and control of assembly pathways. This will provide a theoretical foundation for the on-demand construction of functional materials on the basis of molecular design. However, key challenges remain, including achieving full control from molecular conformation to macroscopic assembly and establishing quantitative relationships between isomer arrangement and function.

Selecting appropriate core building blocks is thus a critical research focus. Traditional organic macrocycles, which are the starting units for positional isomerism, suffer from core defects such as conformational lability and limited responsiveness, resulting in poor controllability of assembly pathways. Coordination-driven self-assembly (CDSA)^8–17^ provides an efficient alternative approach for constructing spatial architectures with well-defined sizes and shapes,^18–26^ which are the ideal building blocks for achieving orders of magnitude improvements in assembly precision^27–35^ and functional dimensionality.^36–42^

Our group reported the successful fabrication of centimeter-scale metallacage-based films through the strategic incorporation of TPE groups into MOCs using PEG-modified metallacages.^43^ To investigate the influence of the initial unit configuration on the hierarchical assembly mechanism, the assembly behaviors of tetraphenylethylene (TPE)-based *meta*- and *para*-positioned MOCs have been systematically studied.^44^ The conformational differences between *meta*- and *para*-positioned MOCs at the Angstrom scale significantly alter their packing modes, resulting in the formation of linear and polyhedral structures, respectively. Meanwhile, the relationship between structures and functions was investigated through pyrene-containing MOCs.^45^ However, the structure–property relationships between these hierarchical assembly structures and material performance have yet to be further clarified.

Here, the positional isomers of metallacycles act as core building blocks, benefiting from the structural rigidity of the metal nodes and the dynamic flexibility of the organic ligands. The integration of positional isomers into MOCs provides precisely controllable molecular building blocks for visualizing assembly pathways, enabling the directional design of complex functional materials. The cross-scale correlation mechanism of configuration–assembly–function can be systematically investigated by the cascade regulatory role of positional isomers in molecular conformational transitions, mesoscale self-assembly, and macroscopic functional emergence.

When 1,1,2,2-tetrakis(4-(pyridin-4-ylethynyl)phenyl)ethene is used as the skeleton and isophthalic acid is used as the linker, platinum coordination yields the *meta*-positioned metallacycle MOC 1. Replacing the linker with terephthalic acid produces the *para*-positioned metallacycle MOC 2. Positional isomers, with their minimal positional differences, selectively participate in hierarchical assembly through flexible variations at specific sites.

The *meta*-positioned MOC 1 forms solid metal–organic materials with ultralong fibrous micro/nanostructures, whereas the *para*-positioned MOC 2 yields only flat ribbon-like structures. X-ray diffraction (XRD) analysis of the MOC 1 structure suggests that in fibrous superstructures, TPE motion is further restricted, effectively suppressing nonradiative transitions. This phenomenon results in blueshifted fluorescence emission and a prolonged fluorescence lifetime. However, the *para*-positioned MOC 2-based ribbon assemblies, leading to redshifted emission and shortened fluorescence lifetimes. These phenomena collectively validate a fundamental principle: the most minute positional differences can trigger macroscopic functional transitions. This principle not only advances fundamental understanding but also opens avenues for designing responsive materials. For instance, MOC 1 fibers, with their enhanced luminescence and stability, could serve as efficient optical waveguides or sensing platforms for environmental pollutants. Conversely, the energy dissipation characteristics of MOC 2 ribbons may be exploited in light-harvesting systems or as quenchers in biosensing applications.

## Results and discussion

**Fig. 1:**
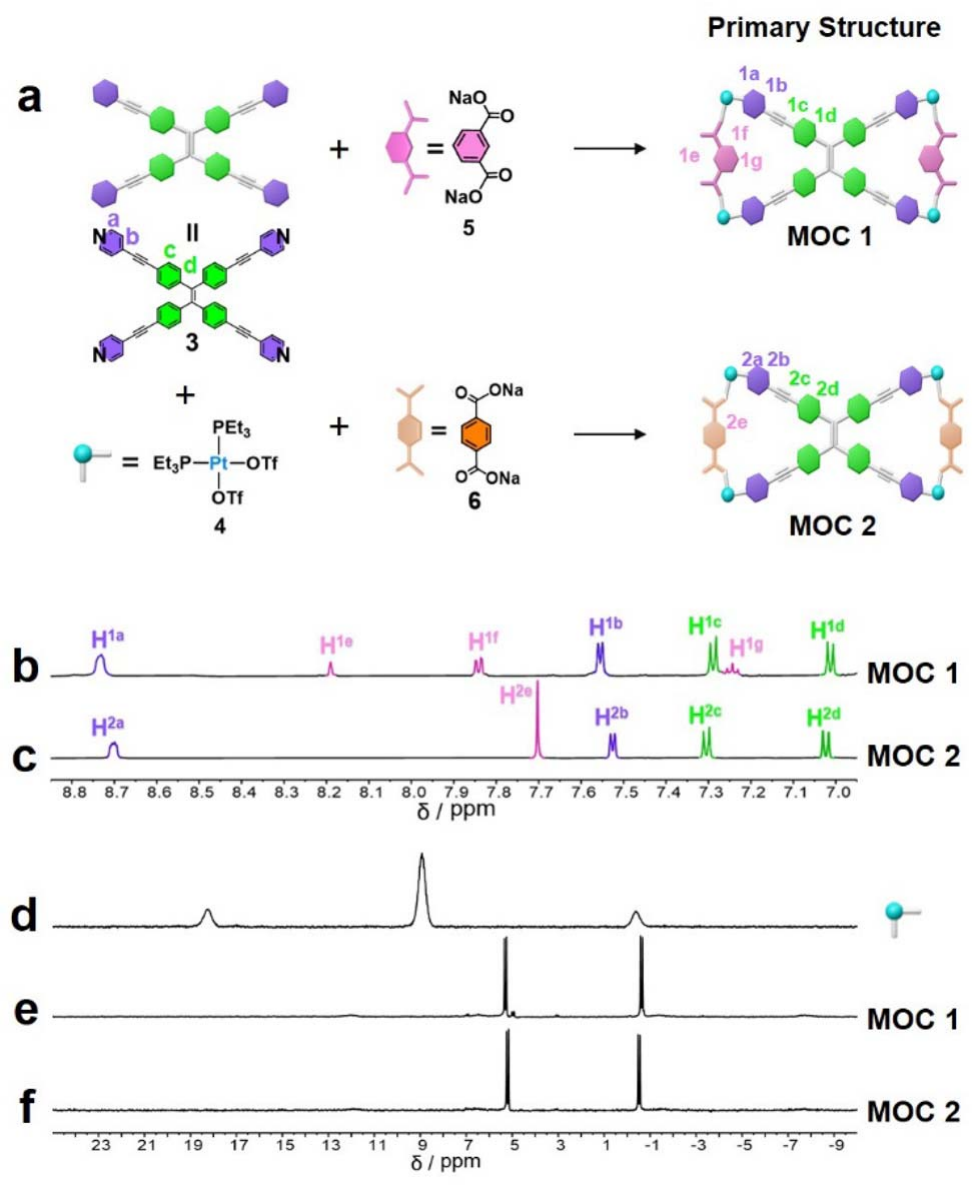
Synthesis of MOCs 1–2 through coordination-driven assembly. (a) [1 + 4 + 2] assembly of 3, 4 and 5 to furnish MOC 1 and of 3, 4 and 6 to furnish MOC 2*via* the heteroligation-directed self-assembly of 90° Pt(ii) acceptors and pyridyl and carboxylate ligands. (b–d) Partial ^1^H NMR spectra of (b) MOC 1, and (c) MOC 2. ^31^P{^1^H} NMR spectra (CD_2_Cl_2_) of (d) 90° Pt(ii) acceptors, (e) MOC 1, and (f) MOC 2 (to distinguish the functional regions in the MOC, the pyridine units are represented in purple, the sections between pyridines are shown in green, the carboxylic acid segments are indicated in pink/orange (*m*-/*p*-), and the platinum functionalities are denoted by the blue region).

As shown in Fig. 1a, MOCs 1–2 were formed from a TPE derivative (3) (Fig. S1), *cis*-(PEt_3_)_2_Pt(OTf)_2_ (4) (Fig. S2 and S3), and dicarboxylate ligands with various carboxylate ligands (5 and 6) (Fig. S4 and S5) through coordination interactions. MOC 1 was prepared by stirring a mixture of TPE (3), a sodium dicarboxylate ligand (5/6) and the 90° Pt(II) acceptor (4) in a 1 : 2 : 4 ratio in an acetone/water (v/v = 8 : 1) mixture. The ^31^P{^1^H} and ^1^H NMR spectra of the reaction mixture suggest the formation of a single, discrete assembly with high symmetry. The ^31^P{^1^H} spectra of MOC 1 show two doublets of approximately equal intensity at *δ* = 5.31 and −0.62 ppm with concomitant ^195^Pt satellite peaks corresponding to two distinct phosphorous environments (Fig. 1e and S7), indicating that the Pt(II) centers on MOC 1 have a heteroligated N, O-coordination motif with one pyridyl and one carboxylate moiety per metal center. In the ^1^H NMR spectrum of MOC 1 (Fig. 1b and S6). The well-defined signals in both the ^31^P{^1^H} and ^1^H NMR spectra indicate that a discrete structure is the sole assembly product (Fig. S6, S7, S9 and S10). The ^31^P{^1^H} spectra of MOC 2 show two doublets of approximately equal intensity at *δ* = 5.21 and −0.52 ppm with concomitant ^195^Pt satellite peaks corresponding to two distinct phosphorous environments (Fig. 1f).

The stoichiometry of discrete MOC 1 is further supported by the electrospray ionization time-of-flight mass spectrometry (ESI-TOF-MS) results. The ESI-TOF-MS spectrum for MOC 1 shows peaks for the assigned [1 + 4 + 2] assembly, including peaks corresponding to an intact entity with charge states resulting from the loss of OTf counterions (*m*/*z* = 979.62 for [M − 3OTf]^3+^) (Fig. S8). The ESI-TOF-MS results for MOC 2 show peaks corresponding to the assigned [1 + 2 + 4] assembly, including peaks corresponding to an intact entity with charge states from the loss of OTf counterions (*m*/*z* = 979.62 for [M − 3OTf]^3+^) (Fig. S11).

To systematically monitor the assembly behavior, spectral comparisons of functional components were conducted to determine characteristic peaks associated with critical functional domains (Fig. S12). On the basis of these comparative experiments, the characteristic absorption peaks corresponding primarily to the following regions were identified.

The UV absorption characteristics of MOC 1 are as follows. The shoulder peak at 290 nm (attributed to the evolution of the peaks of ligand 3 at 283 nm and 298 nm) and the red shift suggest that the conjugated system further expands after coordination. The peak at 317 nm (near the ligand peak at 319 nm, dominated by TPE) largely retains the absorption characteristics of the TPE core, indicating that the π → π* transition of TPE is not strongly perturbed by coordination. The peak at 354 nm (resulting from a blueshift of the ligand peak at 361 nm, dominated by TPE) suggests the changes in the molecular charge distribution after coordination. The more well-defined peak shape (compared with the incomplete peak at 361 nm) suggests a clarified energy level distribution and enhanced transition dipole moments after coordination.

**Fig. 2:**
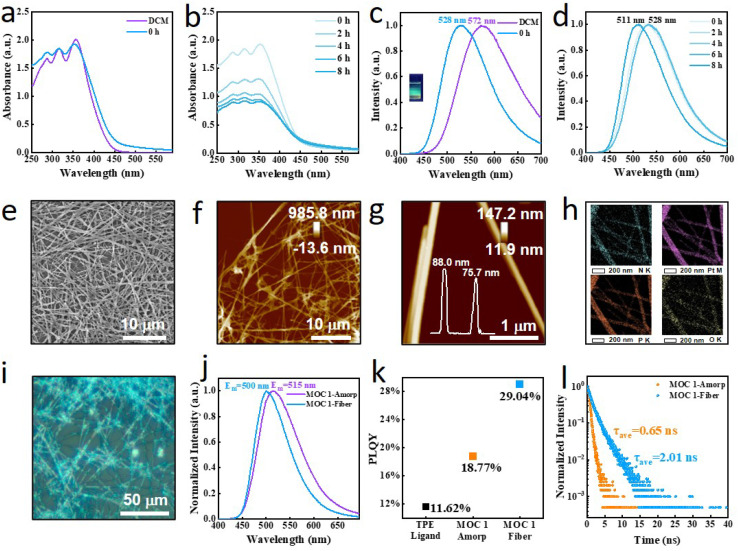
(a) UV-vis spectra of MOC 1 in pure DCM and after the addition of EA (20 μM). (b) Time-dependent evolution of the UV-vis spectrum of MOC 1 in DCM with the addition of EA. (c) Fluorescence spectra of MOC 1 in pure DCM and after the addition of EA. (d) Time-dependent evolution of the fluorescence spectrum of MOC 1 in DCM with the addition of EA. (e) Corresponding SEM image. (f and g) AFM images. (h) EDS mapping image. (i) Polarized light microscopy image of the fibers. (j) Solid-state fluorescence spectra. (k) Photoluminescence quantum yield and fluorescence lifetime of MOC 1 in the amorphous and fibrous state. (l) Fluorescence lifetime of MOC 1 in the amorphous and fibrous states.

Concentration-dependent studies reveal that as the concentration increases from 6 μM to 20 μM, the three absorption peaks remain near 287 nm, 316 nm, and 357 nm, with no significant shifts in their wavelengths; additionally, their absorbance gradually increases with increasing concentration (Fig. S13). Notably, the ratios of the absorbance of these three peaks continuously increase with increasing concentration. At 6 μM, the absorbance ratios among the three peaks are close to 1 : 1 : 1, whereas at 20 μM, the absorbances of the peaks increase progressively from 288 nm to 315 nm to 354 nm. No obvious redshift or blueshift is observed, suggesting that MOC 1 remains monodisperse. The fluorescence spectra show no significant shift, which is consistent with the ultraviolet-visible (UV-vis) results. The UV-vis spectra of MOC 1 in pure dichloromethane and in dichloromethane/ethyl acetate mixtures are shown in Fig. 2a. In pure DCM, the absorbance increases progressively from 287 nm (Abs = 1.67) to 316 nm (Abs = 1.84) to 357 nm (Abs = 2.00). After the addition of ethyl acetate (EA), three absorption peaks are observed at 288 nm (Abs = 1.78), 316 nm (Abs = 1.85), and 353 nm (Abs = 1.92), whereas the ratio of the absorbance values is significantly reduced. In the initial stage (2 hours), multiple aggregation states likely exist, leading to significant differences in absorbance among the different transitions (0 hours/2 hours: 288/288 nm, 316/316 nm, and 353/350 nm). When the assembly time is extended to 8 hours, there is almost no change in peak position (shifts in wavelength), indicating that the electronic transition characteristics do not significantly change (Fig. 2b).

The fluorescence spectra reveal that the introduction of ethyl acetate induces a two-stage blueshift in the fluorescence emission of MOC 1 (Fig. 2c). An initial rapid blueshift of 44 nm (572 → 528 nm) originates from the destabilization of the excited state due to the reduction in solvent polarity. The decrease in solvent polarity (DCM → EA) is the primary reason for the blueshift of the long-wavelength absorption peak and the reduction in the absorbance ratio. This change reflects the modulation of the solvent of the excited state stabilization and transition dipole moments. A subsequent slow blueshift of 17 nm (528 → 511 nm) over 8 hours corresponds to the progressive planarization of the molecular conformation (Fig. 2d). Time-dependent field-emission scanning electron microscopy (FE-SEM) reveals the formation of fibrous structures (Fig. S14).

The FE-SEM image confirms the successful fabrication of continuous fibrous structures (Fig. 2e), which exhibit a homogeneous distribution and well-defined morphology over a wide area. High-resolution transmission electron microscopy (HR-TEM) further supports the formation of fibrous superstructures (Fig. S15a), revealing diameters consistent with those determined by STEM and demonstrating one-dimensional morphological homogeneity. Three-dimensional AFM nanotopography measurements (Fig. 2f and S15b) quantitatively validate the fibrous morphology. The height profile analysis (Fig. 2g) reveals fibers with sizes of 88 nm (point 1) and 76 nm (point 2); these findings are consistent with the SEM and TEM observations.

Elemental mapping analysis *via* energy-dispersive X-ray spectroscopy (EDS) confirms the homogeneous spatial distribution of key constituent elements (Fig. 2h), including nitrogen (N), the characteristic element of tetraphenylethylene (TPE) moieties; platinum (Pt) and phosphorus (P), the signature elements of the *cis*-Pt(PEt_3_)_2_(OTf)_2_ complex; and oxygen (O), which originates from the sodium sulfonate-functionalized carboxylate ligand throughout the microfiber architecture. These results provide direct evidence for the successful coassembly of all three components (TPE, *cis*-Pt(PEt_3_)_2_(OTf)_2_, and the carboxylate ligand) into well-defined composite fibers. Furthermore, the observed elemental distributions, combined with the fiber dimensions and known molecular parameters, suggest that each individual microfiber comprises an ordered supramolecular array of at least several hundred metallacyclic building blocks (Fig. S15c).

Polarized light microscopy (PLM) characterization reveals that the fibers exhibit characteristic blue birefringence interference colors (Fig. 2i), indicating significant optical anisotropy within the fibers. This phenomenon originates from the highly oriented structure within the fibers, demonstrating the successful achievement of highly oriented MOC alignment through self-assembly. Solid-state fluorescence spectroscopy analysis reveals that the fibrous supramolecular assemblies formed through molecular self-assembly present a distinct emission peak at 500 nm, indicating a significant blueshift of approximately 15 nm compared with that of the amorphous preassembled state (emission peak at 515 nm), as shown in Fig. 2j.

Comparative analysis of photoluminescence quantum yield (PLQY) reveals the pronounced regulatory effect of molecular ordered assembly on radiative transition processes: the measurements (using an integrating sphere) demonstrate that the fluorescence quantum yield increases from *Φ*_1_ = 18.77% for the amorphous powder to *Φ*_2_ = 29.04% for the self-assembled fibrous architecture (Fig. 2k). This relative enhancement of 54.7% indicates the effective suppression of nonradiative decay channels. The significant increase in quantum yield (*Φ*: 18.77% → 29.04%) coupled with a prolonged fluorescence lifetime (*τ*: 0.65 → 2.01 ns) collectively demonstrates that self-assembly-induced molecular rigidification and defect passivation synergistically optimize the radiative recombination efficiency (Fig. 2l). In addition, ultralong fibers can be obtained through the controlled assembly of MOC 1 at concentrations of 20 μM, 50 μM and 100 μM (Fig. S16).

**Fig. 3:**
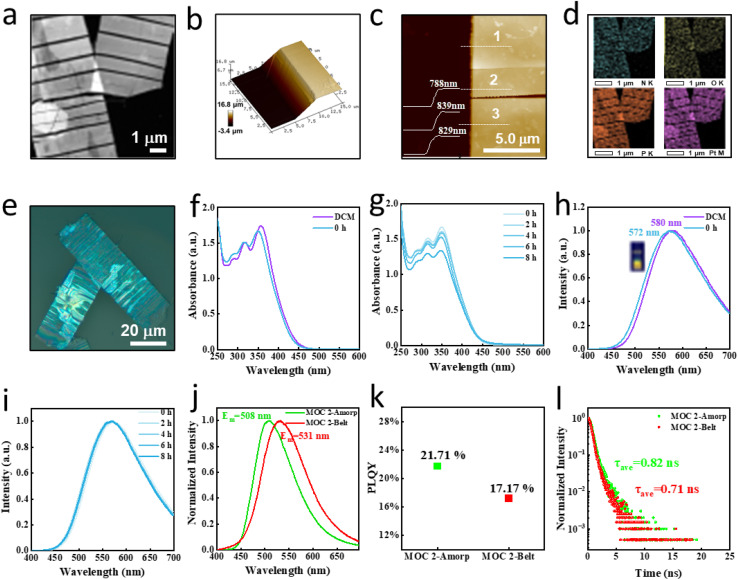
(a) STEM image of the micrometer ribbon. (b and c) Corresponding AFM images. (d) EDS mapping images of the ribbon. (e) Polarized light microscopy image of the ribbon. (f) UV-vis spectra of MOC 2 in pure DCM and after the addition of EA. (g) Time-dependent changes in the parameters of the UV-vis spectra of MOC 2 in DCM after the addition of EA. (h) Fluorescence spectra of MOC 2 in pure DCM and after the addition of EA. (i) Time-dependent changes in the ratio of the fluorescence intensity of MOC 2 in DCM after the addition of EA. (j) Solid-state fluorescence spectra. (k) Photoluminescence quantum yield. (l) Fluorescence lifetime of MOC 2 in amorphous and ribbon-like structures.

As a positional isomer of MOC 1, MOC 2 exhibits markedly distinct assembly characteristics during hierarchical self-assembly. TEM clearly reveals (Fig. 3a) that MOC 2 tends to form flat ribbon structures. This unique morphological feature is in sharp contrast to the well-defined nanofibers formed by MOC 1, fully demonstrating how subtle differences in coordination-induced positional isomerism can significantly influence self-assembly behavior. Notably, TEM shows distinct crack structures on the surfaces of these microribbons. We propose that this morphological characteristic may originate from two primary factors: (1) structural stress induced by interfacial tension during solvent evaporation and (2) an uneven distribution of the synergistic effects between intermolecular π–π interactions and coordination bonds. The formation mechanisms of these crack structures provide important clues for understanding the mechanical properties of coordination supramolecular materials.

The thicknesses of these ribbon-like structures are precisely determined by AFM height profile measurements (Fig. 3b). Cross-sectional analysis of the AFM topographic images reveals that the as-formed ribbons have relatively smooth surfaces with an average thickness of 780 nm, demonstrating good uniformity in the vertical dimension. Systematic characterization of the ribbon-like structures by AFM reveals that these self-assembled ribbons exhibit highly uniform thickness characteristics.

AFM height profile analysis demonstrates that the ribbon surfaces also have good flatness, with an average thickness of 818 nm, confirming the excellent dimensional control in the vertical direction of this self-assembly system. To further investigate the mechanical stabilities of the ribbons, we conducted a detailed AFM characterization of the fractured regions. The measurements revealed that the heights at three different fracture sites were 788 nm, 829 nm, and 839 nm (Fig. 3c). This observation indicates that fracture primarily occurs along the longitudinal axis of the ribbon, but it does not occur through interlayer delamination. The slight variations in thickness observed between different locations may originate from local differences in the molecular packing density. Collectively, these data demonstrate that the microribbons maintain excellent structural homogeneity in both the intact and fractured states, highlighting the remarkable stability of this hierarchical self-assembly system.

EDS elemental mapping clearly reveals the compositional characteristics of the ribbon surfaces. As shown in Fig. 3d, characteristic elements, including Pt, P, N, and O, exhibit highly uniform distribution patterns across the ribbon surfaces. This homogeneous elemental distribution demonstrates that (1) coordination bond formation facilitates uniform self-assembly between metal centers (Pt) and organic ligands (containing P, N, and O); (2) intermolecular interactions (such as hydrogen bonding) enable effective spatial regulation at the nanoscale; and (3) the solvent evaporation process does not induce significant elemental segregation. Particularly noteworthy is the uniform distribution of Pt, which provides direct evidence for the ordered arrangement of metal nodes within the assembly. Furthermore, the correlation between the elemental distribution and AFM morphological characteristics provides additional verification of the chemical homogeneity of the ribbon structure. POM reveals that the ribbon structures exhibit remarkable light emission properties (Fig. 3e).

To investigate the molecular basis of the differences in assembly behavior, we conducted studies using UV-vis spectroscopy (Fig. S17). In pure dichloromethane, three absorption peaks are observed at 292 nm, 318 nm, and 357 nm. After introducing ethyl acetate, the peak shifts are almost negligible (less than 2 nm). The prolonged assembly time does not significantly affect the peak positions at 318 nm and 293 nm (shifts less than 2 nm), whereas the peak at 358 nm undergoes a blueshift to 347 nm. After 8 hours of assembly, three peaks are observed at 294, 315, and 347 nm (a blueshift of 10 nm). In comparison, the blueshift occurs from 358 nm to 347 nm. Fluorescence spectroscopy reveals that the emission peak in pure dichloromethane is located at 580 nm. After the addition of ethyl acetate, the system blueshifts to 572 nm. Over the subsequent 8 hours, no further shift is observed (Fig. 3i), with the emission peak remaining at 572 nm. We subsequently compared the emission spectra before assembly with those of the ribbon-like structure and found that the preassembly emission peak occurs at 508 nm, whereas the ribbon structure presented a redshifted emission peak at 531 nm (a shift of 23 nm). Along with the redshift in fluorescence emission, the quantum yield significantly decreased from 21.71% to 17.17%.

**Fig. 4:**
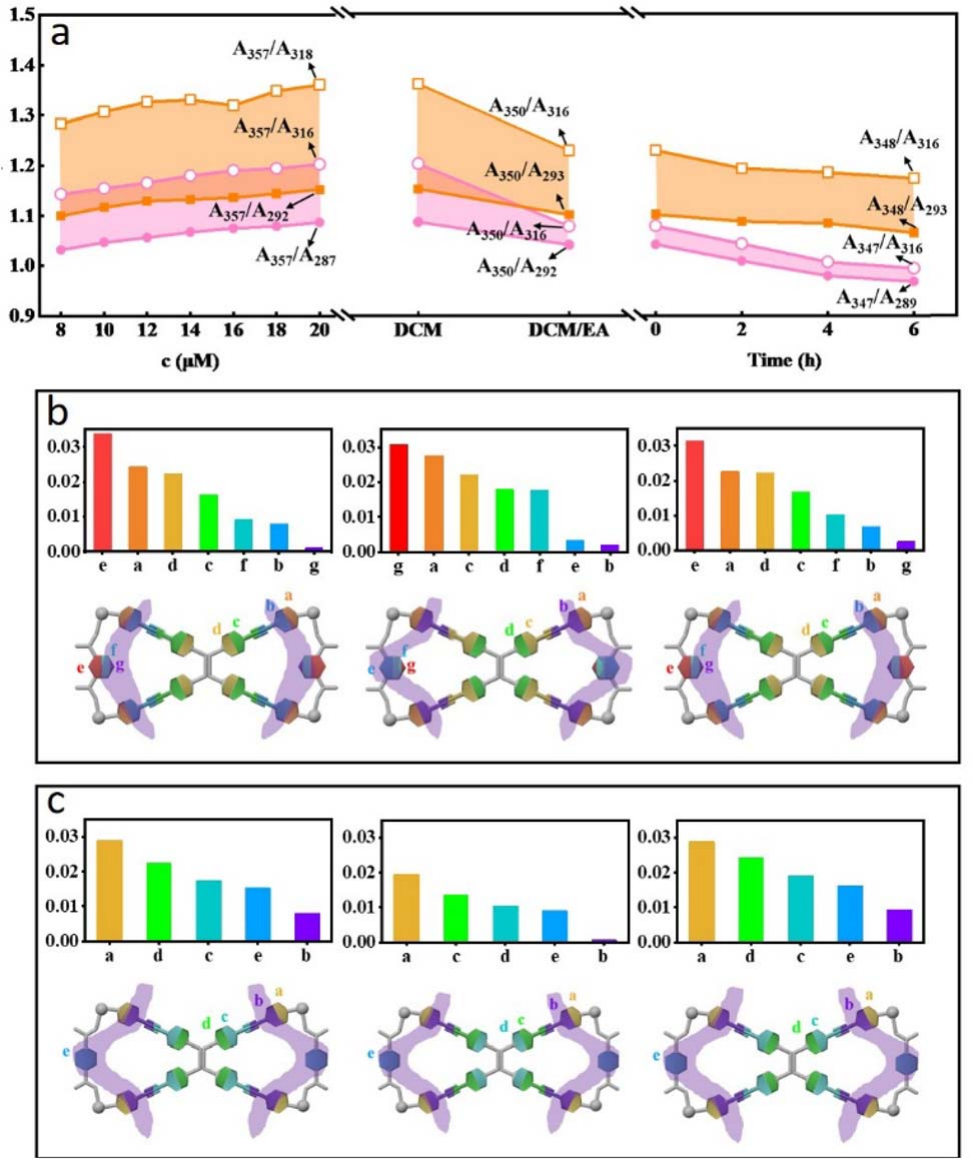
(a) *A*_357_/*A*_316_ and *A*_357_/*A*_287_ ratios in pure DCM with increasing concentrations of MOC 1 after EA addition and the time-dependent dotted line plots in DCM/EA. (b) Differences in the chemical shifts of MOC 1 in dichloromethane between −10 °C and 30 °C (left); in DCM and DCM/EA (middle) (20 °C); and between −10 °C and 30 °C (right). (c) Differences in the chemical shifts of MOC 2 in dichloromethane between −10 °C and 30 °C (left); in DCM and DCM/EA (middle); and in DCM/EA between −10 °C and 30 °C (right).

UV-vis spectroscopic analysis of MOC 1 was used to track its evolution under three distinct conditions, revealing time-dependent assembly behavior (Fig. 4a). In pure DCM, increasing the MOC 1 concentration from 8 to 20 μM resulted in a progressive increase in the *A*_357_/*A*_316_ (1.11 to 1.20) and *A*_357_/*A*_287_ (1.01 to 1.09) ratios, indicating an increased contribution from the metal coordination center (Fig. 4a, left). The introduction of EA to the solution (20 μM) of MOC 1 markedly decreased both ratios (*A*_357_/*A*_316_ from 1.20 to 1.08; *A*_357_/*A*_287_ from 1.09 to 1.04), suggesting solvent-mediated structural reorganization but maintenance of the integrity of the metal coordination core (Fig. 4a, middle).

Time-resolved studies in the DCM/EA mixed solvent system demonstrated sequential spectral changes: the initial ratios of *A*_357_/*A*_316_ and A_357_/A_287_ (1.08/1.04) decreased after 2 hours (1.04/1.01), approached unity at 4 hours, and ultimately fell below 1 by 6 hours (Fig. 4a, right). This temporal evolution provides clear evidence for kinetically controlled assembly processes induced by changes in the solvent microenvironment. Comparative analysis with MOC 2 revealed conserved assembly trends and distinct structural outcomes (Fig. 4a, orange line). Both systems showed concentration-dependent ratio increases that are characteristic of monomeric states, and the solvent-induced ratio decreased upon EA addition. However, MOC 2 maintained higher *A*_357_/*A*_318_ ratios (∼1.2) after 6 hours than MOC 1, suggesting preserved spectral differentiation between coordinated and non-coordinated moieties.

Complementary NMR studies elucidated the molecular-scale dynamics (Fig. 4b and c). For MOC 1 in DCM, reducing the temperature (30 °C to −10 °C) induced the largest changes in chemical shift (Fig. S19) for the carboxylic (H^e^) and pyridyl (H^a^) protons, with TPE protons showing intermediate flexibility (Fig. 4b left). EA introduction initially enhanced carboxylic (H^g^) proton dynamics (Fig. S20) while maintaining pyridyl (H^a^) and TPE flexibility (Fig. 4b middle, Fig. S20). Strikingly, variable-temperature studies (Fig. 4c middle, Fig. S21) in the binary solvent system revealed the carboxylic (H^e^) and pyridyl (H^a^) shows largest changes. The most active regions were marked as purple shadow. MOC 2 exhibited different dynamic signatures, with pyridyl (H^a^) protons remaining the most flexible in both pure DCM (Fig. 4c left, Fig. S22) and under the initial EA conditions (Fig. 4c middle, Fig. S23). The binary solvent system again induced dynamic reorganization, with H^a^ and H^d^ to be the most mobile sites in the assembled state (Fig. 4c right, Fig. S24). These comprehensive spectroscopic analyses establish that carboxylic and pyridyl segments make significant contributions to the assembly process of the isomers assembly pathways.

Although the crystal structure reveals the presence of hydrogen bonding interactions such as C=O⋯H–C, π–π stacking and counterion effects may also play synergistic roles in the assembly process. Future selective perturbation experiments, such as systematically varying the solvent polarity (to modulate the hydrogen bond strength), introducing functional group substitutions (to precisely disrupt specific hydrogen bonds), or replacing counterions, will provide more direct and causal evidence for the relative contributions of various interactions.

**Fig. 5:**
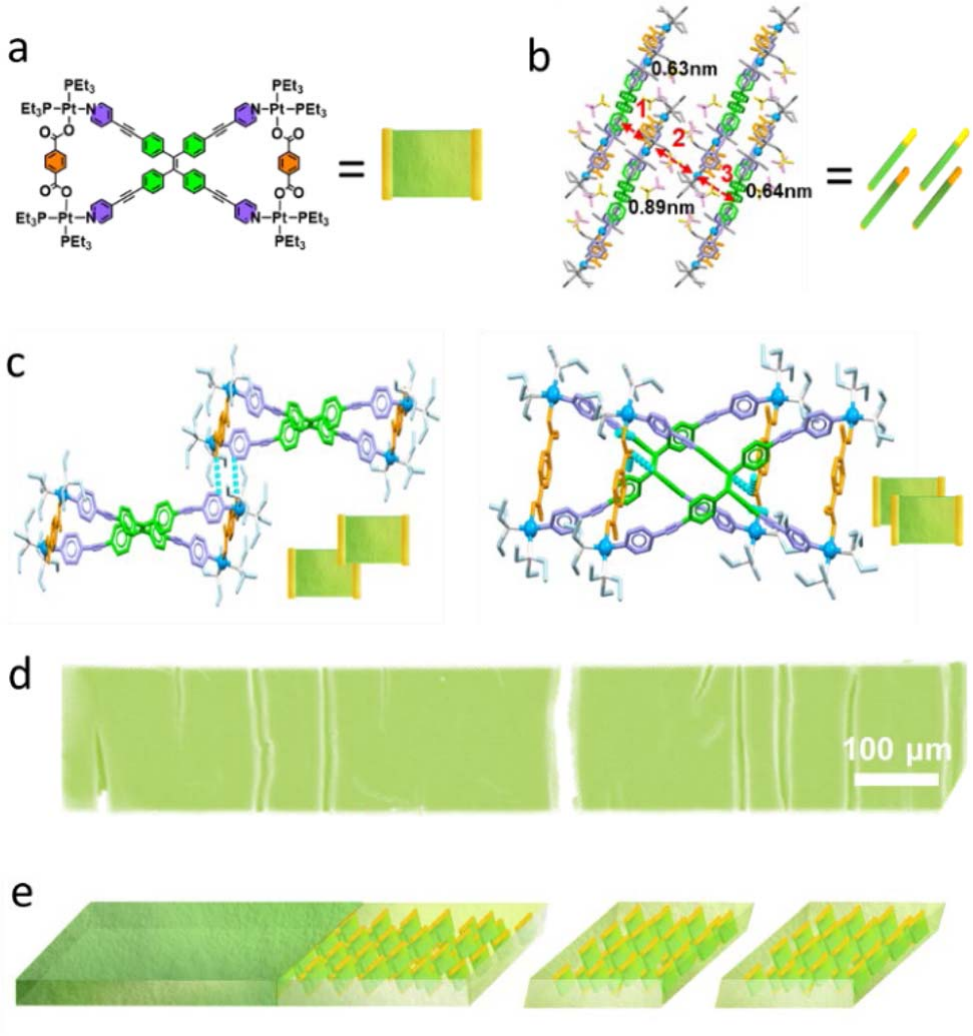
(a) Molecular structure of MOC 2. (b) Packing mode of MOC 2. (c and d) Critical interactions between two MOCs. (d) SEM image of the micrometer ribbons. (e) Stacking mode of MOC 2 in the micrometer ribbons.

The XRD analysis of the MOC 2 ribbons revealed a set of characteristic diffraction peaks (0.89 nm, 0.64 nm, 0.63 nm, 0.48 nm, and 0.46 nm) in the low-angle region (Fig. S25) compared with those of the amorphous powder (Fig. S26). Combined with single-crystal structure determination (Fig. 5b, S27–S29, Tables S2 and S3), these peaks confirm the existence of periodic layered arrangements along the *x*-axis with spacings of 0.89 nm, 0.64 nm, and 0.63 nm. This structural ordering originates from two types of intermolecular interactions: (1) primary hydrogen bonding (C=O⋯H–C) between the carbonyl oxygen of carboxylic acid groups (acting as hydrogen bond acceptors) and H–C on adjacent pyridine rings (Fig. 5c, left) and (2) secondary C–H⋯O=C hydrogen bonding, where the carboxylic carbonyl groups interact weakly with C–H bonds (H–C_sp_^2^) from TPE units (Fig. 5c, right). These C–H⋯O=C interactions play a significant role in directing molecular packing, lead to the formation of microribbons (Fig. 5d).

Notably, the 0.48 nm and 0.46 nm diffraction peaks likely correspond to the π–π stacking distances between molecular planes. The small difference in spacing reflects slight anisotropy in molecular packing. These spacings (in the range of 0.45–0.50 nm) indicate relatively loose π–π stacking, potentially resulting from the significant steric hindrance due to the rotating phenyl rings in TPE units, preventing tight packing. By integrating the XRD structural analyses, DFT calculations, and electron microscopy results, we propose the following molecular self-assembly mechanism. The C=O groups of terephthalic acid form interlocking hydrogen bonds with the pyridyl C–H and TPE phenyl ring C–H of adjacent molecules, driving the parallel staggered arrangement of MOC 2 and their extension along a two-dimensional plane. Since hydrogen bonding is significantly stronger than the van der Waals forces in the vertical direction, the structure preferentially grows within this plane. Moreover, the staggered orientation of the molecules further determines the preferred growth axis of the ribbon-like assembly. These complementary forces collectively guide preferential molecular growth along specific crystallographic directions, ultimately yielding highly ordered supramolecular ribbon structures (Fig. 5e).

The differences in the assembly behavior of MOCs are primarily attributed to their molecular structures (Fig. S31). For MOC 1 (Fig. S32, S33, Tables S4 and S5), the assembled fibers (Fig. S34) exhibit more characteristic diffraction peaks (1.02 nm, 0.91 nm, 0.83 nm, and 0.72 nm) than the XRD data before assembly (Fig. S35), suggesting the formation of additional complex superstructures.

**Fig. 6:**
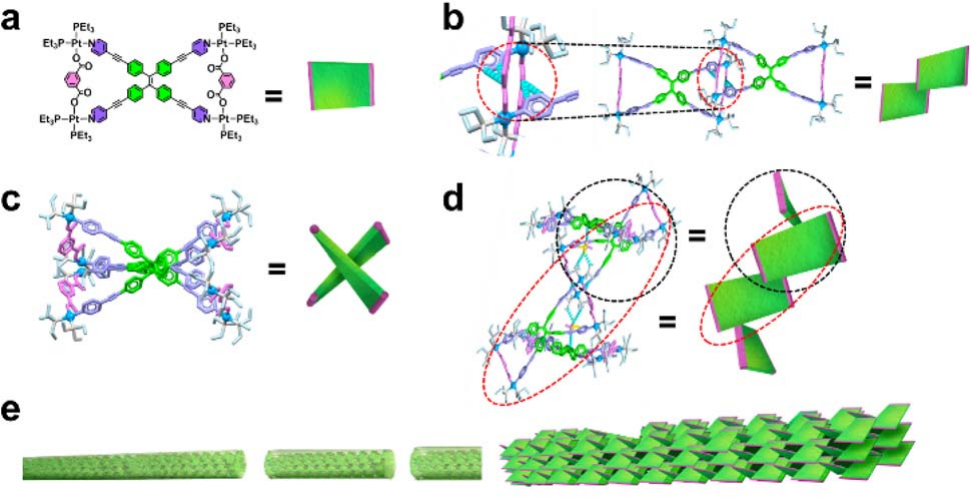
(a) Molecular structure of MOC 1. (b and c) Critical interactions between two MOCs. (d and e) Packing mode of MOC 1.

Due to differences in the symmetry of MOC 1 (Fig. 6a), intermolecular hydrogen bonding exhibits angular deviations, whereas variations in trifluoromethanesulfonate-mediated hydrogen bonding interactions lead to an intercrossed stacking arrangement of adjacent *meta*-positioned molecular planes (Fig. 6b and c). This configuration forms an interlocked and stable fibrous surface along the planar direction, facilitating growth oriented parallel to the MOC 1 plane (Fig. 6d and e). Ultimately, this trend results in the assembly of fibrous structures with high aspect ratios and uniform fibers.

The self-assembly behavior of MOC 1 was also investigated in DCM/hexane systems, and fibrous structures were consistently obtained (Fig. S31a). Similarly, when the assembly behaviors of MOC 2 in DCM/hexane systems were studied, ribbon-like structures were observed (Fig. S31b).

**Fig. 7:**
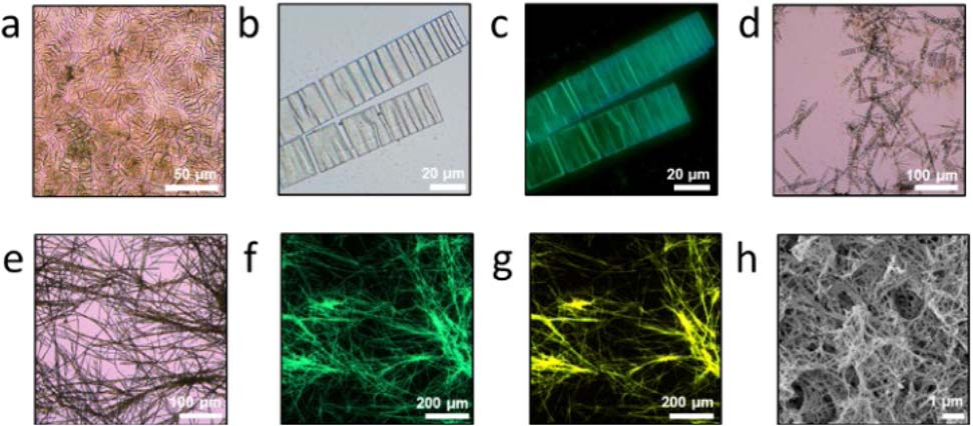
(a and b) Different magnifications of OM image of the micrometer ribbon (MOC 2 in DCM/Dio), (c) FOM image of the micrometer ribbon with UV (340–380 nm) light (MOC 2 in DCM/Dio), (d) OM image of the micrometer ribbon (MOC 2 in DCM/*n*-pentane), (e) OM image of the fibers based (MOC 1 in DCM/*n*-pentane), (f) FOM image of the fibers with UV (340–380 nm) light (MOC 1 in DCM/*n*-pentane), (g) FOM image of the fibers with UV (450–490 nm) light (MOC 1 in DCM/*n*-pentane), (h) SEM image of fibers (MOC 1 in DCM/*n*-hexane).

Variation in the solvent ratio did not affect the formation of these dominant architectures. MOC 2 can form micrometer-scale ribbon structures through assembly in both DCM/Dio (5/5) (Fig. 7a–c) and DCM/*n*-pentane (5/5) (Fig. 7d) systems (50 μM), whereas MOC 1 assembles into micrometer-scale fibrous structures in both DCM/*n*-pentane (5/5) (Fig. 7e–g) and DCM/*n*-hexane (5/5) (Fig. 7h) systems.

**Fig. 8:**
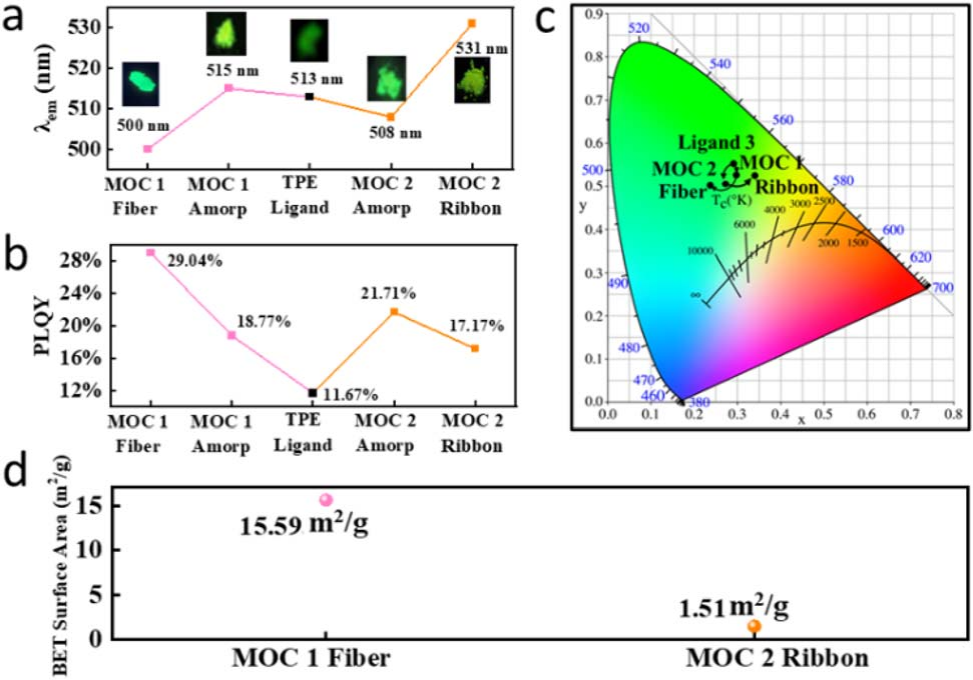
(a) Influences of coordination and further assembly on solid-state emissions, corresponding (b) quantum yield and (c) CIE diagram, (d) specific surface areas of MOC 1-based nanofibers and MOC 2-based micrometer ribbons.

The influence of positional isomerism on molecular packing modes primarily manifests in distinct variations in optical properties. As illustrated in Fig. 8a, the solid TPE ligand exhibits an emission maximum at 513 nm. Upon coordination, *meta*-MOC 1 has an emission peak at 515 nm, whereas the para-bicyclic analog exhibits an emission maximum at 508 nm. Further molecular assembly induces characteristic spectral shifts: MOC 1 displays a blueshift from 515 nm (amorphous state) to 500 nm after fiber formation, whereas MOC 2 exhibits a redshift from 508 nm to 531 nm, which is indicative of energy dissipation processes. Comparative analysis of photoluminescence quantum yield (*Φ*) values between amorphous and hierarchical states reveals structure–property correlations. The *meta*-isomer MOC 1 shows an increase in *Φ* from 18.77% (amorphous) to 29.04% (fibrous crystalline form), which is consistent with its hypsochromic shift. Conversely, the *Φ* of the *para*-isomer MOC 2 decreases from 21.71% to 17.17% upon the formation of micrometer-scale ribbons, which accounts for its bathochromic shift of approximately 23 nm (Fig. 8b). The radiative (*k*_r_) and non-radiative (*k*_nr_) decay rate constants were calculated,^46^ showing that *k*_nr_ for MOC 1 fibers decreased by 72% compared with that of the amorphous powder, offering quantitative support for the “suppression of non-radiative decay” and the “rigidification effect”. In contrast, *k*_nr_ increased by 22% for MOC 2 ribbons, confirming “energy dissipation” as the dominant excited-state decay pathway.

The similar trends are observed in other assemblies formed in different solvents system, which is consistent with those measured in DCM/EA systems. As shown in Fig. S37, the quantum yield and fluorescence lifetime of the MOC 1 fibrous superstructure assembled in the DCM/Hex solution are 25.02% and 1.43 ns, respectively. The quantum yield and fluorescence lifetime of the MOC 2 ribbon superstructure assembled in the DCM/Hex solution system are 19.88% and 0.74 ns, respectively.

In addition to the optical properties, the molecular packing mode significantly influences the specific surface area. A representative example shows that the *para*-isomeric MOC 2 forms micrometer-scale ribbon structures with a specific surface area of only 1.51 m^2^ g^−1^, whereas the *meta*-isomeric MOC 1 assembles into ultralong fibrous structures exhibiting an order of magnitude increase in specific surface area (15.6 m^2^ g^−1^). These findings provide further evidence for the direct impact of molecular *meta*-/*para*-isomerism on the performance of hierarchically assembled architectures (Fig. 8d).

## Conclusions

Through the precise positional control of *meta*-donors *versus para*-donors in metallacycles, we establish a paradigm demonstrating the mechanisms by which minimal structural modifications drive substantial functional divergence in supramolecular systems. We demonstrate that metal-coordinated macrocyclic positional isomers serve as ideal building blocks for hierarchical self-assembly, where minimal structural variations—such as *meta*-substitution *vs. para*-substitution—translate into dramatic differences in assembly pathways and functional outcomes. By leveraging the rigid yet tunable characteristics of metal–organic suprastructures, we establish a precise configuration–assembly–function correlation, revealing the mechanisms through which positional isomerism govern molecular flexibility, mesoscale organization, and macroscopic properties. Specifically, *meta*-isomers enable hypsochromic-shifted emissions and order of magnitude increases in the surface areas of fibrous assemblies. Conversely, *para*-isomers lead to bathochromic-shifted emissions and limited surface areas in ribbon structures, directly correlating the isomer configurations with optoelectronic and interfacial properties.

## Materials and methods

All reagents were commercially available and used as supplied without further purification. Deuterated solvents were purchased from Aladdin, Macklin, and TCI. Compounds 3, 4, 5, and 6 were prepared according to modified procedures detailed in the literature. ^1^H NMR spectra and ^31^P{1H} NMR spectra were recorded in the designated solvents using Bruker 500 MHz spectrometer and Quantum-I Plus 600 MHz spectrometer. The variable-temperature NMR spectra were recorded on Quautum-I Plus 600 MHz spectrometer. The TEM investigations were performed with a JEOL JEM-F200 instrument. For TEM, dispersions of the assemblies were dried on carbon-coated copper support grids. For SEM, dispersions of the assemblies were dried on silicon wafers, and the investigations were performed with a JEOL JSM-7610F Plus instrument. Absorption and fluorescence emission spectra were recorded on Shimadzu UV-2600i and Hitachi F-4700 spectrophotometers, respectively. Specific surface area data were obtained from Micromeritics Instrument Co. ASAP2460 and measured after 8 hours of degassing at 60 °C. Fluorescence microscopy investigations were performed with a Leica DM2700M instrument. Single-crystal data were deposited with CCDC number 2474172, 2486534.

## Author contributions

Y. Sun designed the general experiments. Y. Sun designed the MOC as the primary building blocks for further assembly strategies and superstructure-dependent property experiments; L. Liu performed the experiments; L. Liu and Y. Zhao analyzed the single-crystal data; L. Liu, F. Zhang, and W. Tuo analyzed the NMR data; L. Liu analyzed the mass spectra and fluorescence data; M. Yu analyzed the microscopy data and drew a scheme; L. Liu, Y. Sun wrote the paper.

## Conflicts of interest

There are no conflicts to declare.

## Supplementary Material

SC-016-D5SC05591A-s001

SC-016-D5SC05591A-s002

## Data Availability

CCDC 2474172 and 2486534 contain the supplementary crystallographic data for this paper.^47*a*,*b*^ The data supporting this article have been included as part of the supplementary information (SI). Supplementary information: additional NMR, mass spectra, UV-vis, and fluorescence spectra data, scanning electron microscope, transmission electron microscopy, and AFM were provided in the SI. See DOI: https://doi.org/10.1039/d5sc05591a.
