# Supplementary material for: Carboxylate positional isomerism in metallacycles governs hierarchical assembly pathways
Source: Chem Sci. 2025 Oct 13;16(46):21887–96. doi: 10.1039/d5sc05591a (PMC12536309; doi:10.1039/d5sc05591a)
Supplement: SC-016-D5SC05591A-s001 [file SC-016-D5SC05591A-s001.pdf]

Supplementary Information (SI) for Chemical Science.
This journal is © The Royal Society of Chemistry 2025

Carboxylate Positional Isomerism in Metallacycles Governs Hierarchical Assembly Pathways

Lingran Liu [a], Meilin Yu [a], Wei Tuo[b], Yue Zhao[c], Fengmin Zhang[d], Yan Sun[a,*]

a School of Nanoscience and Materials Engineering, Henan University, Zhengzhou 450046, China

b. Department of Chemistry, University of Utah, 315 South 1400 East, Room 2020, Salt Lake City, Utah 84112, United States

c. School of Chemistry and Chemical Engineering, Nanjing University, Nanjing, 210093, P. R. China

d. Testing Center of Yangzhou University, Yangzhou, Jiangsu, 225002, China

Email: elaine.sun@henu.edu.cn

Supporting Information

1. Materials and methods 4
2. Characterization of MOC 1-2 5
2.1 Synthesis of MOC 1....5
2.2 Synthesis of MOC 2....5
2.3 ^{1}H NMR spectrum of Ligand 3 6
2.4 ^{1}H NMR spectrum of compound 4 6
2.5 ^{31}P {^{1}H} NMR spectrum of compound 4 7
2.6 ^{1}H NMR spectrum of ligand 5....7
2.7 ^{1}H NMR spectrum of ligand 6....8
2.8 ^{1}H NMR spectrum of MOC 1....8
2.9 ^{31}P {^{1}H} NMR spectrum of MOC 1....9
2.10 ESI -TOF-MS spectrum of MOC 1 9
2.11 ^{1}H NMR spectrum of MOC 2....10
2.12 ^{31}P {^{1}H} NMR spectrum of MOC 2....10
2.13 ESI -TOF-MS spectrum of MOC 2 11
3. Self-assembly of MOC 1-2 11
3.1 UV–vis spectra of precursors and MOC 1-2 in DCM....11
3.2 UV-vis and fluorescence spectra of MOC 1 at different concentrations in DCM........13
3.3 Time-dependent SEM images of MOC 1 13
3.4 STEM and AFM images of MOC 1 13
3.5 SEM images and corresponding size histogram of MOC 1-based self-assemblies......14
3.6 UV–vis and Fluorescence spectra of MOC 2 at different concentrations in DCM 15
3.7 SEM images of MOC 2-based self-assemblies....15
4. Partial ^{1}H-NMR spectrum of MOC 1-2 16
4.1 Partial Variable-temperature ^{1}H-NMR spectrum of MOC 1 in CD_2Cl_2....16
4.2 Partial ^{1}H NMR spectrum of MOC 1 in CD_2Cl_2 and CD_2Cl_2/EA mixtures....16
4.3 Partial variable-temperature ^{1}H NMR spectrum of MOC 1 in CD_2Cl_2/EA 17
4.4 Partial variable-temperature ^{1}H NMR spectrum of MOC 2 in CD_2Cl_2....17
4.5 Partial ^{1}H NMR spectrum of MOC 2 in CD_2Cl_2 and CD_2Cl_2/EA mixtures....18
4.6 Partial variable-temperature ^{1}H-NMR spectrum of MOC 2 in CD_2Cl_2/EA....18
4.7 The XRD pattern of MOC 2 ribbon 18
4.8 The XRD pattern of MOC 2 amorphous powders....19
4.9 X-ray single-crystal structure of MOC 2 19
4.10 Stacking mode of MOC 2-based self-assemblies 19
4.11 Crystallographic Data for MOC 2....20
4.12 Intermolecular hydrogen bonds of MOC 2....22
4.13 The binding configurations of ribbon planes and organization of distinct dimer configurations in MOC 2 assemblies. 22
4.14 Microscopy image of MOC 1-2 assemblies in DCM/Hexane....23
4.15 X-ray single-crystal structure of MOC 1 23
4.16 Stacking mode of MOC 1-based self-assemblies 23
4.17 Crystallographic Data for MOC 1....24

4.18 The XRD pattern of MOC 1 fibers 25
4.19 The XRD pattern of MOC 1 amorphous powders 26
4.20 Interaction energies and energy decomposition for MOC 2 26
4.21 Fluorescence lifetime images of MOC 1-2 assemblies in DCM/Hexane 27
Reference 28

1. Materials and methods

All reagents were commercially available and used as supplied without further purification. Deuterated solvents were purchased from Aladdin、Macklin、TCI、Energy Chemical. Compounds 3, 4, 5, and 6 were prepared according to modified procedures detailed in the literature. ^{1}H NMR spectra and $^{31}P\{^{1}H\}$ NMR spectra were recorded in the designated solvents using Bruker 500 MHz spectrometer and Quantum-I Plus 600 MHz spectrometer. The variable-temperature NMR spectra were recorded on Quautum-I Plus 600 MHz spectrometer. The TEM investigations were performed with a JEOL JEM-F200 instrument. For TEM, dispersions of the assemblies were dried on carbon-coated copper support grids. HRTEM images were obtained using a JEOL JEM-F200 instrument. For SEM, dispersions of the assemblies were dried on silicon wafers, then the SEM investigations were performed with a JEOL JSM-7610F Plus instrument. Absorption and fluorescence emission spectra were recorded on a Shimadzu UV-2600i and Hitachi F-4700 Spectrophotometer, respectively. A Hitachi F-4700 fluorescence spectrophotometer was used to obtain fluorescence spectra of the sample-free control (using an Al_2O_3 white plate as a reference) and samples (including the sample powder) both directly and indirectly with excitation at 365 nm. All of these data were input into the quantum yield calculation software to obtain φd (quantum yield upon direct excitation), Ad (absorbance upon direct excitation), and φi (quantum yield upon indirect excitation). The quantum yield was calculated after correcting for the influence of indirect excitation using the following equation: $\varphi = \varphi d - (1\text{-}Ad)\ \varphi i$. Specific surface area dates were obtained from Micromeritics Instrument Co. ASAP2460 and measure at a degassing temperature of 60°C for 8 hours. Fluorescence microscope investigations were performed with a Leica DM2700M instrument. A suitable crystal was selected and on a Bruker D8 VENTURE TXS PHOTON II diffractometer. The crystal was kept at 193.00 K during data collection. Using Olex2 [1], the structure was solved with the SHELXT [2] structure solution program using Intrinsic Phasing and refined with the SHELXL [3] refinement package using Least Squares minimisation.

1. Dolomanov, O.V., Bourhis, L.J., Gildea, R.J, Howard, J.A.K. & Puschmann, H. (2009), J. Appl. Cryst. 42, 339-341.
2. Sheldrick, G.M. (2015). Acta Cryst. A71, 3-8.
3. Sheldrick, G.M. (2015). Acta Cryst. C71, 3-8

2. Characterization of MOCs

2.1 Synthesis of MOC 1

A solution of 1,1,2,2-tetrakis(4-(pyridin-4-ylethynyl)phenyl)ethene **3** (3.75mg, 2.5 μmol) in 3 mL acetone was mixed with a solution of carboxylate ligand **5** (2.10 mg, 5.0 μmol) in 2 mL H_2O, the resulting mixture was heated and stirred at 50 °C for 5 min, the solution will be clear, then, the acetone solution of cis-$Pt(PEt_3)_2(OTf)_2$ **4** (14.6 mg, 10.0 μmol) was added into the mixture. Afterwards, the reaction mixture was allowed to slowly cool to room temperature, after stirring for 8 h, the solvent was removed by a flow. The resultant residue was dissolved in 2 mL DCM, and then filtered to remove insoluble materials. The filtrate was concentrated using flow to afford **MOC 1**, light yellow powder, yield: 93%. The ^{1}H NMR spectrum of **MOC 1** is shown in **Figure S6**. ^{1}H NMR (600 MHz, CD_2Cl_2, room temperature) δ (ppm): 8.75-8.71(m, 8H), 8.19 (s, 2H), 7.85 (d, J = 7.80 Hz, 4H), 7.56 (d, J = 6.00 Hz, 8H), 7.30 (d, J = 7.80 Hz, 8H), 7.26-7.23 (m, 2H), 7.02 (d, J = 8.40 Hz, 8H), 1.92-1.86 (m, 24H), 1.79-1.73 (m, 24H), 1.33-1.27 (m, 72H). The ^{31}P {^{1}H} NMR spectrum of **MOC 1** is shown in **Figure 7**. ^{31}P {^{1}H} NMR (243 MHz, CD_2Cl_2, room temperature) δ (ppm): 5.31 (d, $J_{P\text{-}P}$ =21.6Hz, ^{195}Pt satellites, $^1J_{Pt\text{-}P}$=3239 Hz), -0.62 (d, $J_{P\text{-}P}$ = 21.6 Hz, ^{195}Pt satellites, $^1J_{Pt\text{-}P}$=3430 Hz). The stoichiometry of the MOCs is further supported by the electrospray ionization time of the flight mass spectrometry (ESI-TOF-MS) results. ESI-TOF-MS (m/z): calcd for $[M\text{–}3OTf]^{3+}$, 979.62; found 979.62 **(Figure S8)**.

2.2 Synthesis of MOC 2

A solution of 1,1,2,2-tetrakis(4-(pyridin-4-ylethynyl)phenyl)ethene **3** (3.75 mg, 2.5 μmol) in 3 mL acetone was mixed with a solution of carboxylate ligand **6** (2.10 mg, 5.0 μmol) in 2 mL H_2O, the resulting mixture was heated and stirred at 50 °C for 5 min, the solution will be clear, then, the acetone solution of cis-$Pt(PEt_3)_2(OTf)_2$ **4** (14.6 mg, 10.0 μmol) was added into the mixture. Afterwards, the reaction mixture was allowed to slowly cool to room temperature, after stirring for 8 h, the solvent was removed by a flow. The resultant residue was dissolved in 2 mL DCM, and then filtered to remove insoluble materials. The filtrate was concentrated using flow to afford **MOC 2** light yellow powder, yield: 93%. The ^{1}H NMR spectrum of **MOC 2** is shown in **Figure S9**. ^{1}H NMR (600 MHz, CD_2Cl_2, room temperature) δ (ppm): 8.73-8.67 (m, 8H), 7.70 (s, 8H), 7.53 (d, J=5.40 Hz, 8H), 7.31 (d, J=8.40 Hz, 8H), 7.03 (d, J=8.40 Hz, 8H),1.94-1.86 (m, 24H), 1.80-1.72 (m, 24H), 1.31-1.26 (m, 72H). The ^{31}P {^{1}H} NMR spectrum of **MOC 2** is shown in **Figure S10**. ^{31}P{^{1}H} NMR (243 MHz, CD_2Cl_2, room temperature) δ (ppm): 5.18 (d, $J_{P\text{-}P}$ =21.6 Hz, ^{195}Pt satellites, $^1J_{Pt\text{-}P}$=3310 Hz), -0.54 (d, $J_{P\text{-}P}$ = 21.6 Hz, ^{195}Pt satellites, $^1J_{Pt\text{-}P}$=3415 Hz). ESI-TOF-MS (m/z): calcd for $[M\text{–}3OTf]^{3+}$, 979.62; found 979.62 **(Figure S11)**.

2.3 ^{1}H NMR spectrum of compound 3

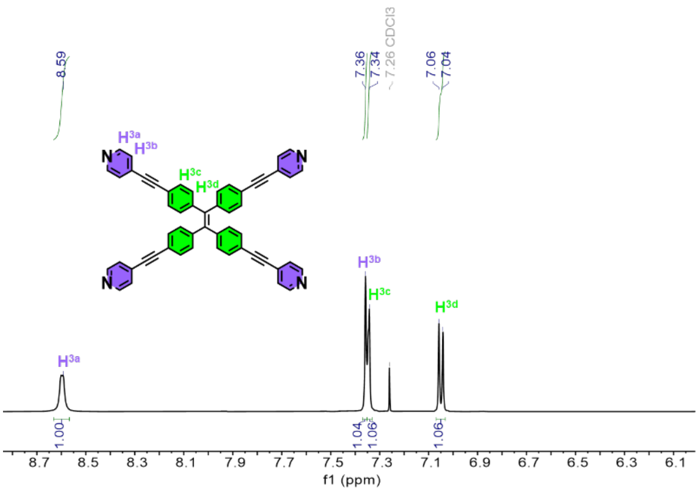

Figure S1. ^{1}H NMR spectrum (500 MHz, $CDCl_3$) recorded for ligand 3.

2.4 ^{1}H NMR spectrum of compound 4

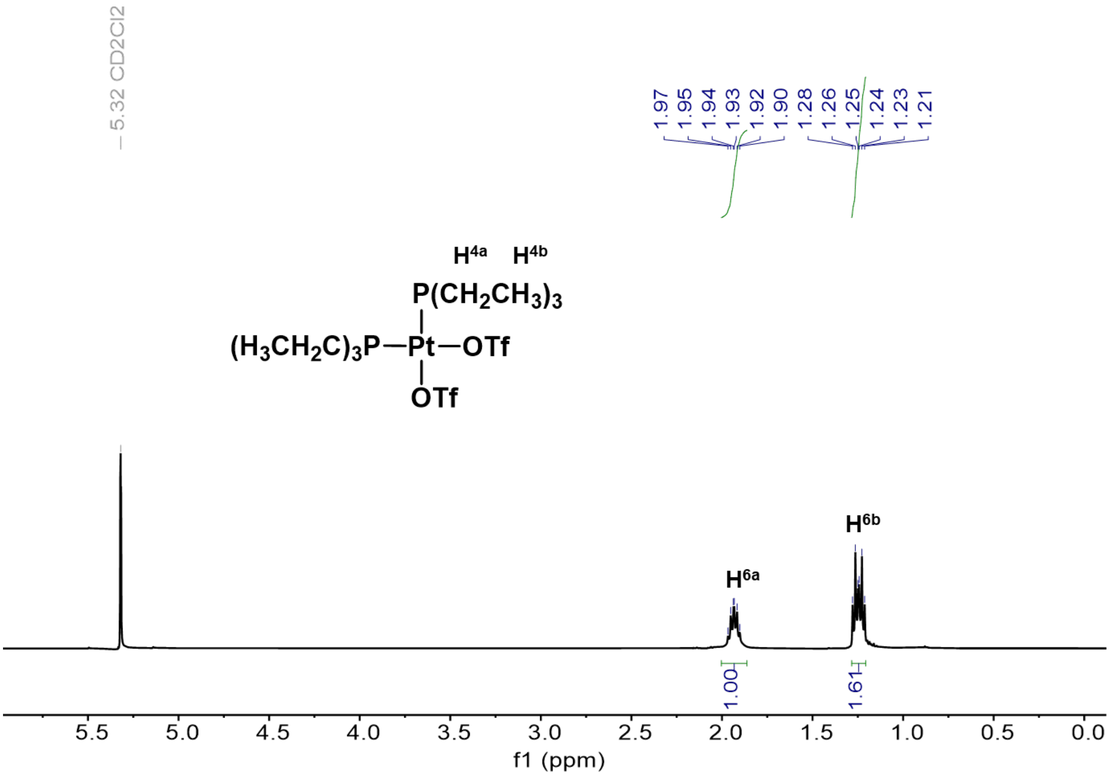

Figure S2. ^{1}H NMR spectrum (500 MHz, CD_2Cl_2) recorded for **4**.

2.5 $^{31}P\{^{1}H\}$ NMR spectrum of compound 4

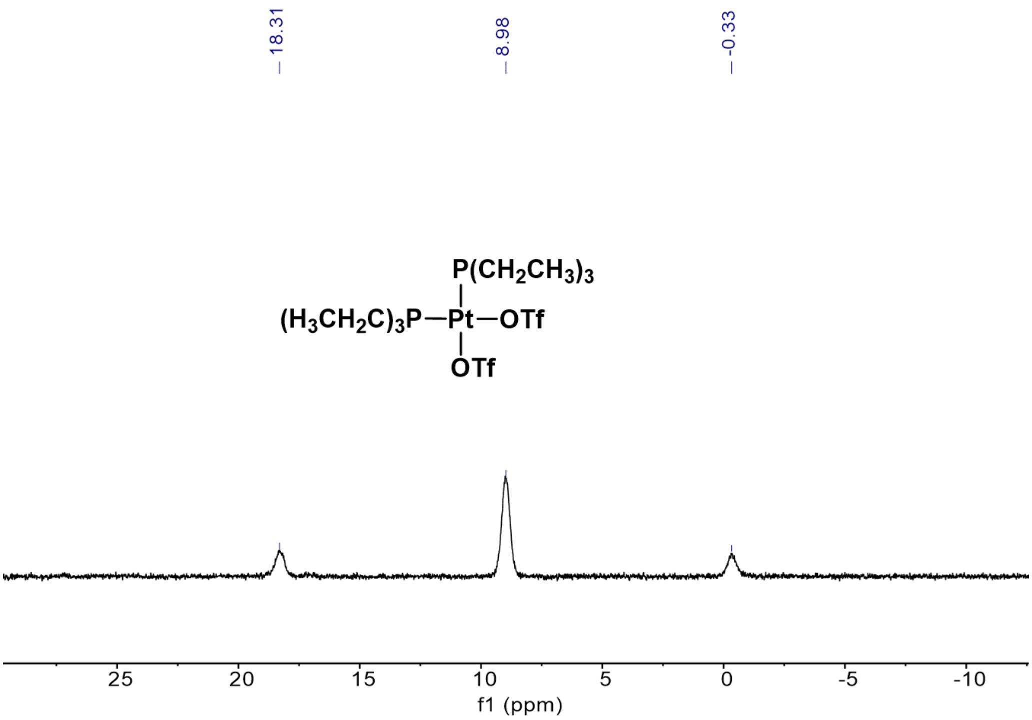

Figure S3. $^{31}P\{^{1}H\}$ NMR spectrum (202 MHz, CD_2Cl_2) recorded for **4**.

2.6 ^{1}H NMR spectrum of ligand 5

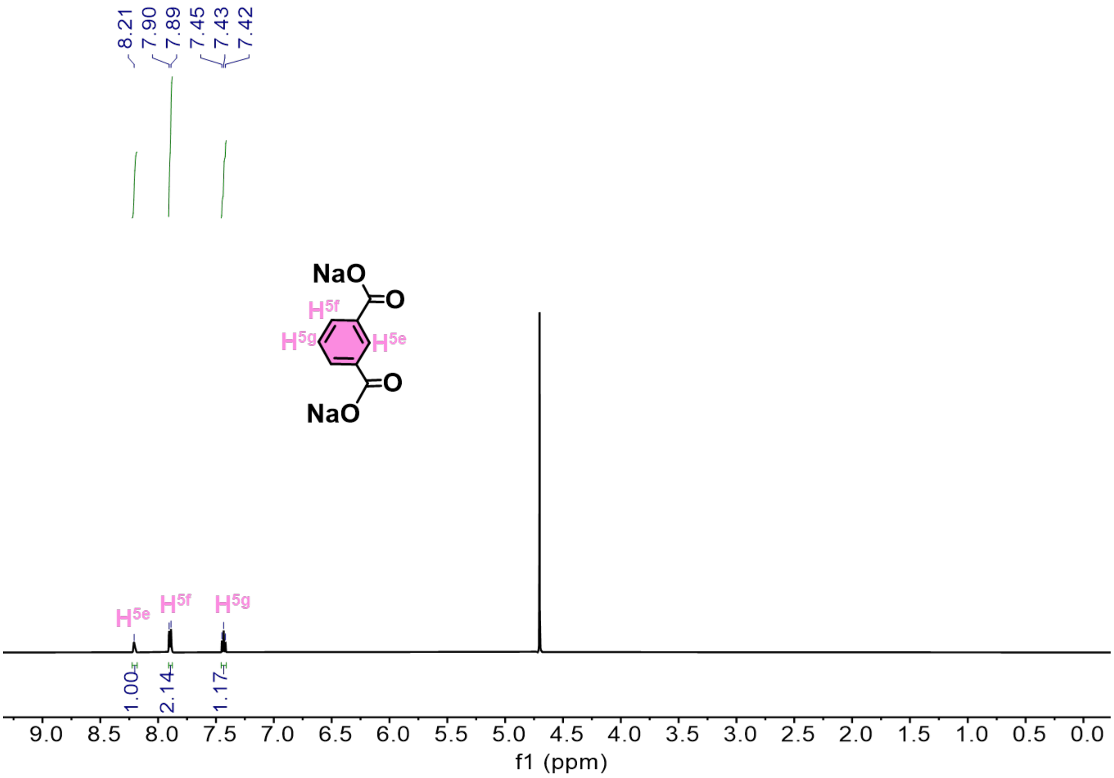

Figure S4. ^{1}H NMR spectrum (500 MHz, D_2O) recorded for ligand 5.

2.7 ^{1}H NMR spectrum of ligand 6

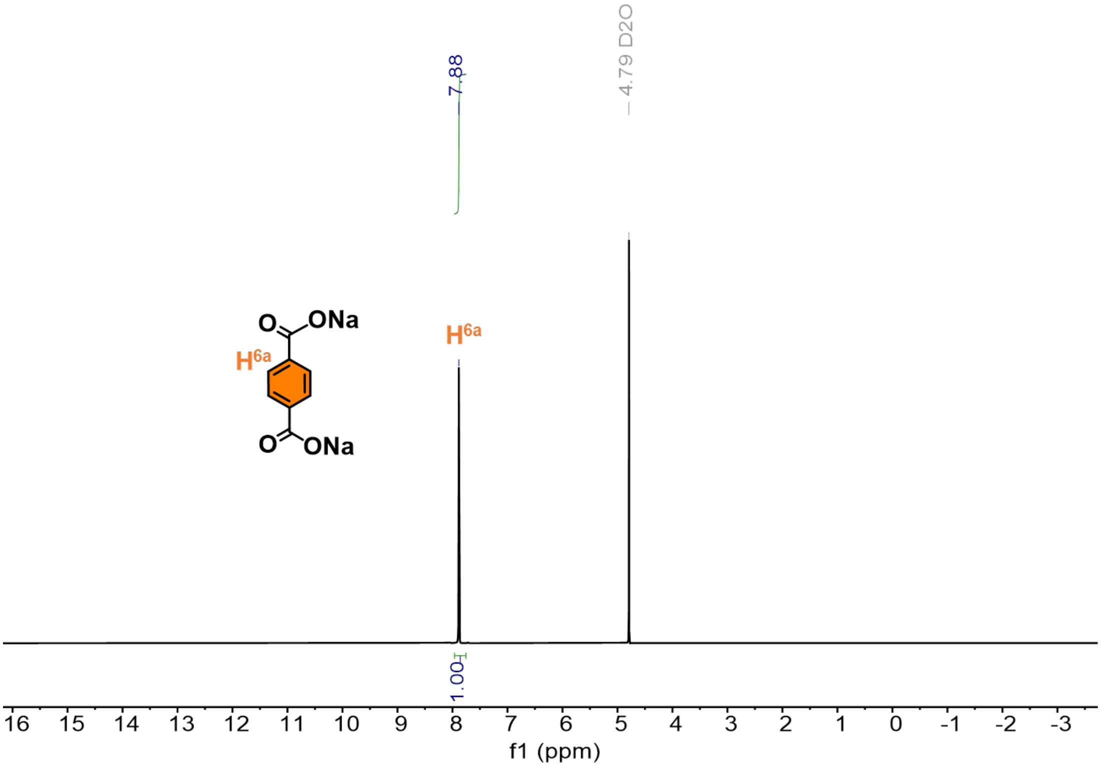

Figure S5. ^{1}H NMR spectrum (500 MHz, D_2O) recorded for ligand 6.

2.8 ^{1}H NMR spectrum of MOC 1

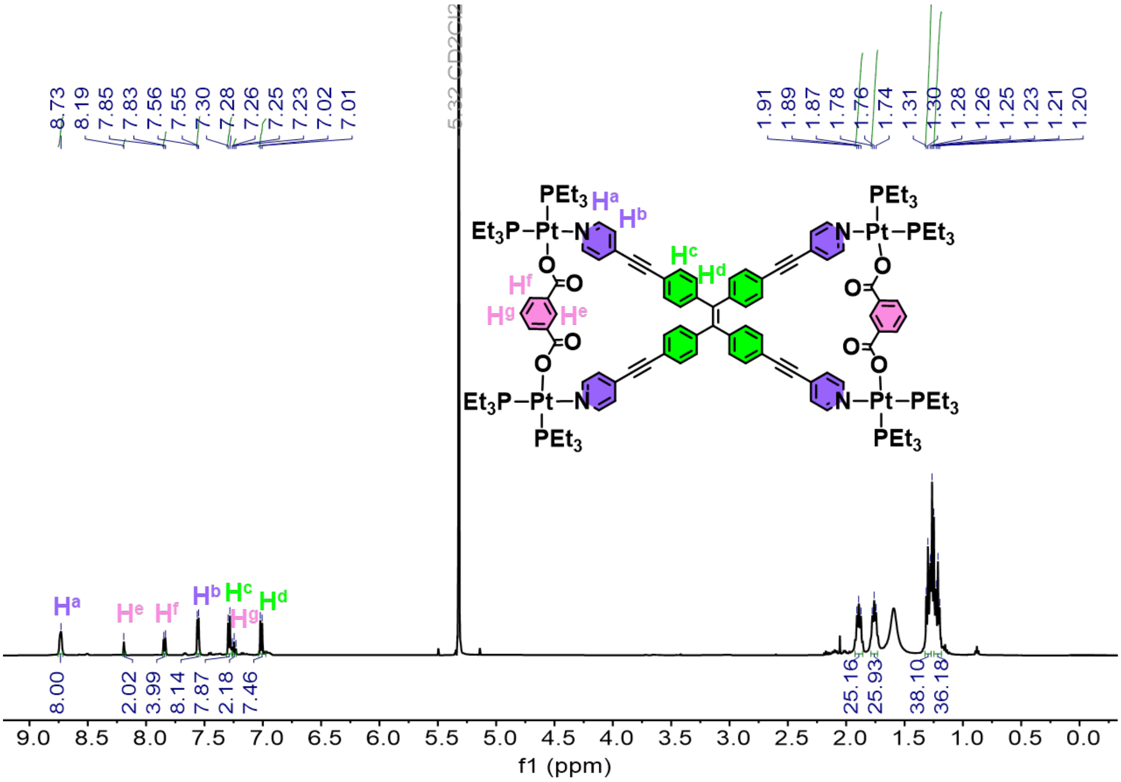

Figure S6. ^{1}H NMR spectrum (600 MHz, CD_2Cl_2) of **MOC 1**.

2.9 $^{31}P\{^1H\}$ NMR spectrum of MOC 1

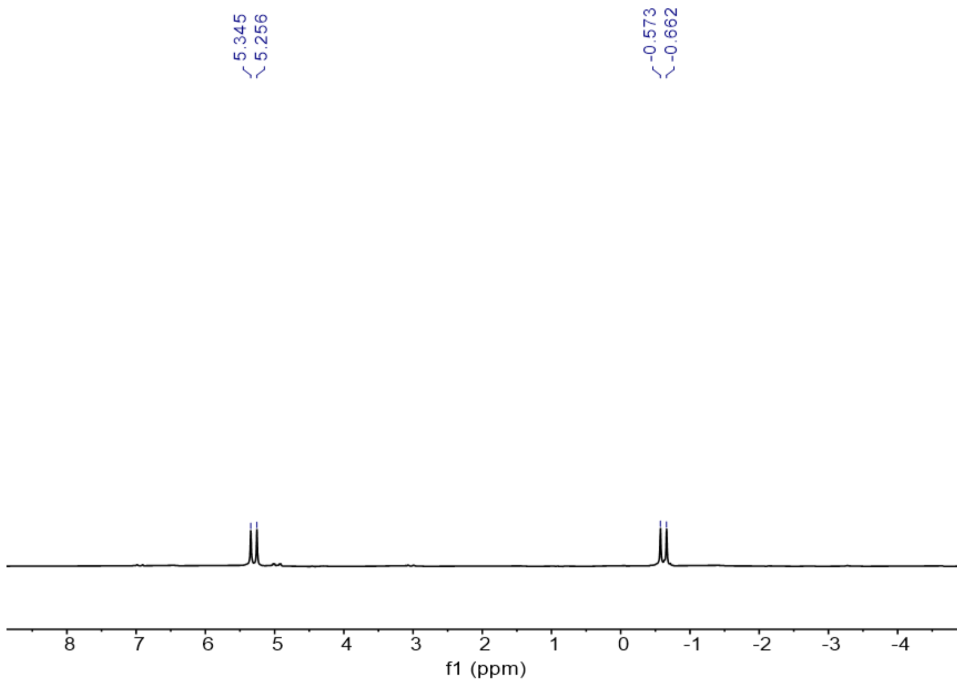

Figure S7. $^{31}P\{^1H\}$ NMR spectrum (243 MHz, CD_2Cl_2) of **MOC 1**.

2.10 ESI -TOF-MS spectrum of MOC 1

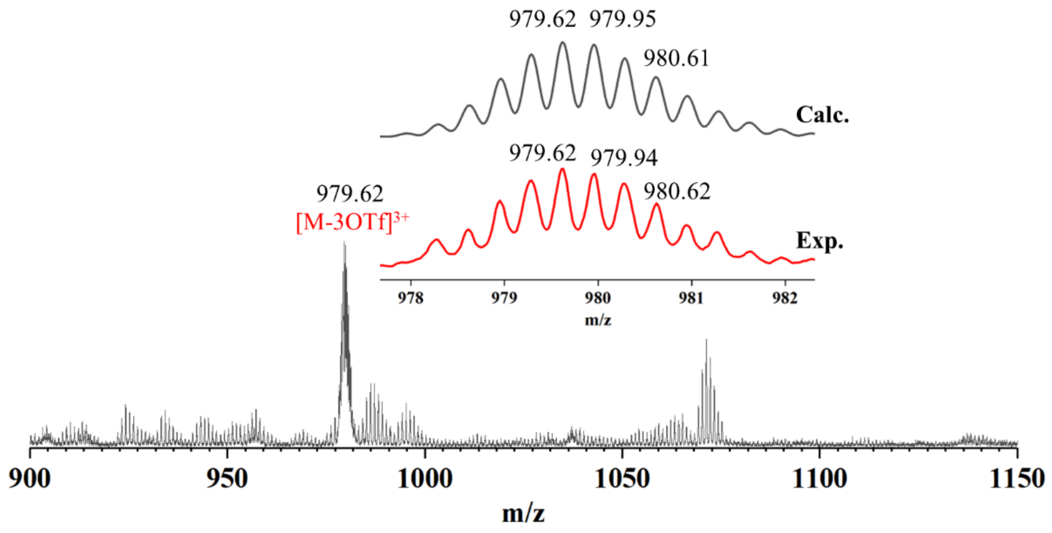

Figure S8. ESI-TOF-MS spectrum of **MOC 1**.

2.11 ^{1}H NMR spectrum of MOC 2

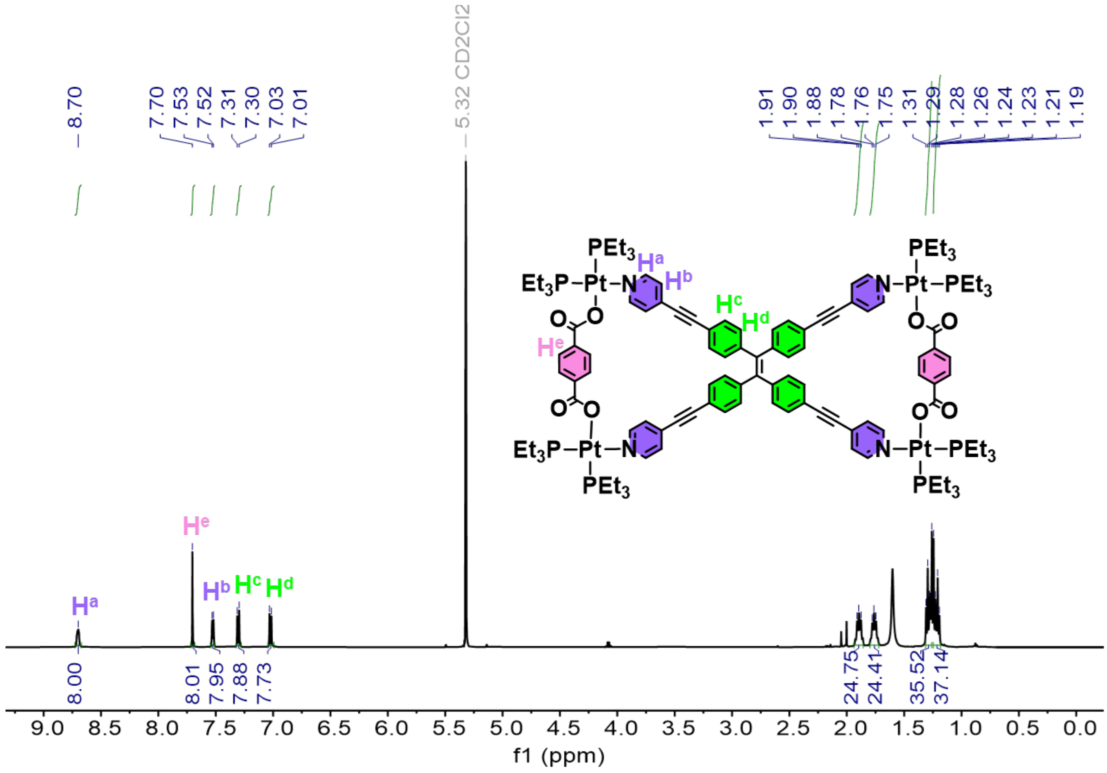

Figure S9. ^{1}H NMR spectrum (600 MHz, CD_2Cl_2) of **MOC 2**.

2.12 ^{31}P {^{1}H} NMR spectrum of MOC 2

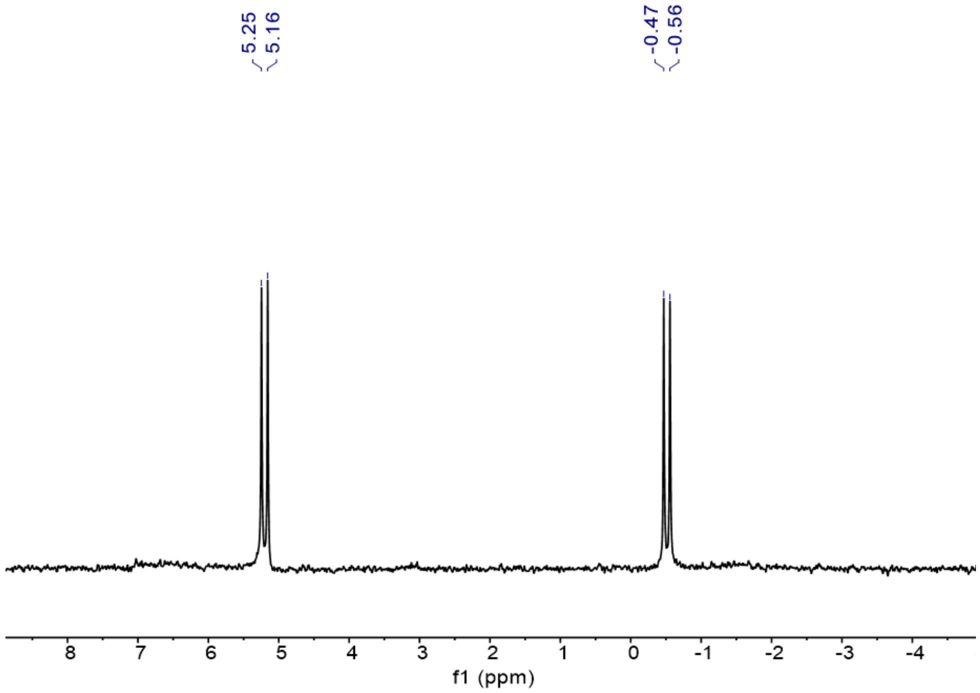

Figure S10. ^{31}P {^{1}H} NMR spectrum (243 MHz, CD_2Cl_2) of **MOC 2**

2.13 ESI -TOF-MS spectrum of MOC 2

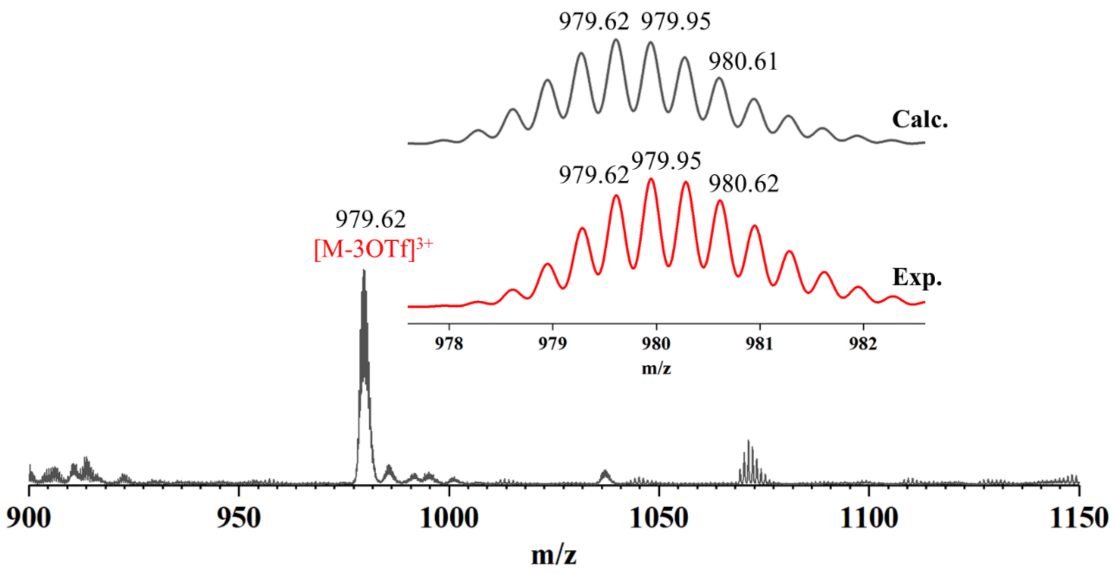

Figure S11. ESI-TOF-MS spectrum of **MOC 2**.

3. Self-assembly of MOC 1-2

3.1 UV–vis spectra of precursors and MOC 1-2 in DCM

The UV spectrum of tetrapyridyl TPE exhibits three absorption peaks at 277 nm, 309 nm, and 340 nm. For the tetrapyridyl TPE with alkyne groups, the UV absorption peaks appear as four, located at 283 nm, 298 nm, 319 nm, and 361 nm. Comparing the UV spectra of the tetrapyridyl TPE, it can be observed that in the alkyne-substituted tetrapyridyl TPE, the conjugated effect of the alkyne groups slightly extends the π-system of the pyridine rings, causing the characteristic pyridine peak to redshift from 277 nm to 283 nm. The peak at 298 nm may arise from the cooperative transition of the benzene-alkyne-pyridine conjugated system. The absorption at 319 nm results from the redshift of the 309 nm peak (a characteristic absorption peak of TPE), while the peak at 361 nm originates from the redshift of the 340 nm peak (associated with the large conjugated structure of TPE and pyridine).

The redshift phenomenon is likely due to the introduction of alkyne groups, which extend the π-conjugated system. The conjugation between the benzene rings, alkyne groups, and pyridine enhances the overall delocalization of the molecule, reducing the energy gap for $\pi \rightarrow \pi^*$ transitions

and leading to the redshift of the absorption peaks.

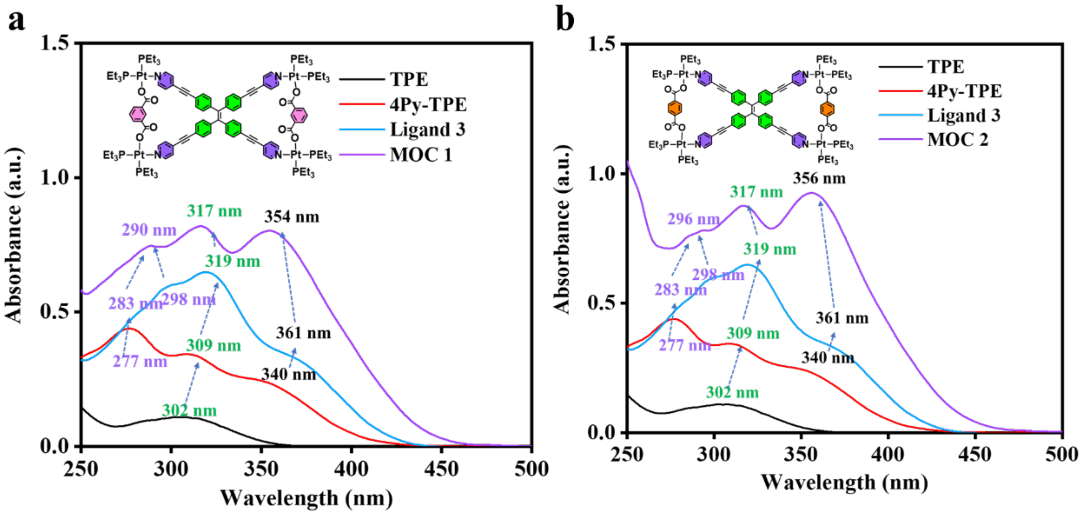

Figure S12. UV–vis of precursors and (a) **MOC 1**, (b) **MOC 2** in DCM (10 μM).

Table S1. Experimental and TD-DFT Calculated Absorption Maxima (λ_{max}), Oscillator Strengths (*f*), Major MO Transitions, Contributions, and Assignments.

Exp. λ_{max} (nm)	Calc. λ_{max} (nm)	State	Oscillator Strength (*f*)	Major MO Transitions	Contribution (%)	Assignment
~358	367.7	S1	0.775	574a → 575a	72.4	Intraligand π→π*
~316	326	S2	2.48	574a → 576a	42.6	Intraligand π→π*
				573a → 575a	26	
	305.2	S3	2.309	574a → 577a	32	Intraligand π→π*
~287	288.7	S8	0.067	574a → 578a	22.1	Intraligand π→π*
				573a → 576a	20.1	

The molecular geometry was first optimized in the ground state using the B97-3c composite method [1], and the nature of the stationary point was confirmed as a true minimum via frequency calculations. Subsequently, the electronic absorption spectrum was simulated using Time-Dependent Density Functional Theory (TD-DFT). These calculations were performed employing the range-separated hybrid functional ωB97X-D3 [2, 3] and the def2-SV(P) basis set [4] within the conductor-like polarizable continuum model (CPCM) for dichloromethane. All calculations were carried out using the ORCA 6.0.1 program package [5]. To elucidate the nature of the observed transitions, the resulting molecular orbitals were analyzed and visualized using the Multiwfn program [6, 7]. The theoretical analysis indicates that the photophysical properties of MOC in the UV-vis region are determined by the electronic structure of the π-conjugated ligand.

3.2 UV−vis and fluorescence spectra of MOC 1 at different concentrations in DCM.

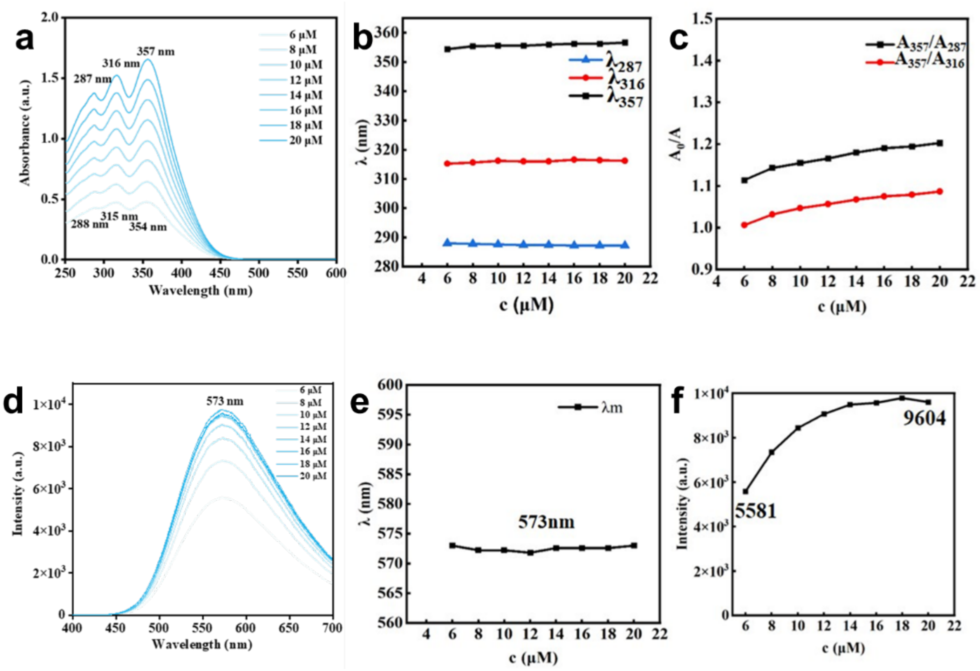

Figure S13. (a) UV–vis spectra of **MOC 1** in DCM (6-20 μM), (b) relationships between **λ** and concentrations, (c) the ratio of A_{357}/A_{287} change with concentration increases. (d) fluorescence spectra of **MOC 1** in DCM (6-20 μM), (e) relationships between λ_{em} and concentrations, (f) the fluorescence intensity with concentration increases.

3.3 Time-dependent SEM images of MOC 1

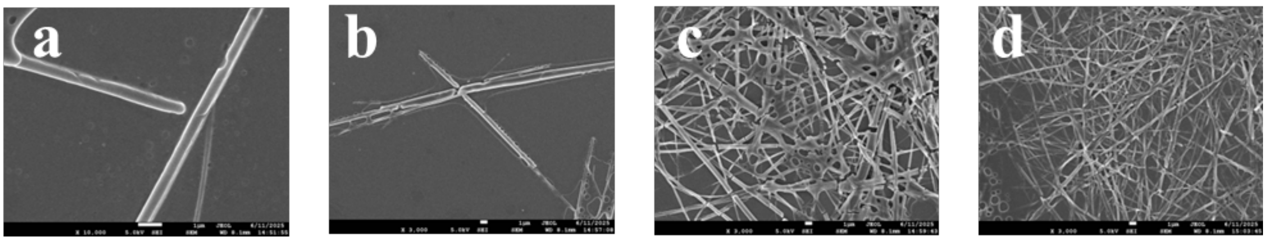

Figure S14. Time-dependent SEM images of (a) 2h, (b) 4h, (c) 6h, (d) 8h.

3.4 STEM and AFM images of MOC 1

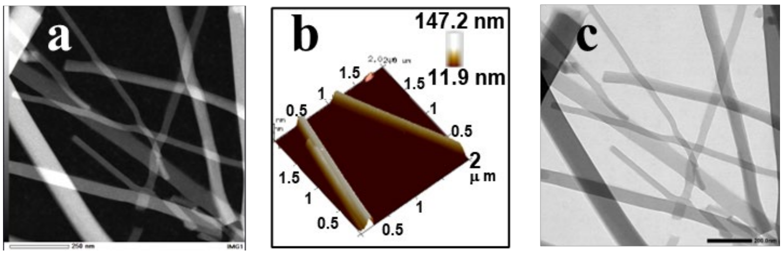

Figure S15. STEM and AFM images of MOC 1.

3.5 SEM images and corresponding size histogram of MOC 1-based self-assemblies

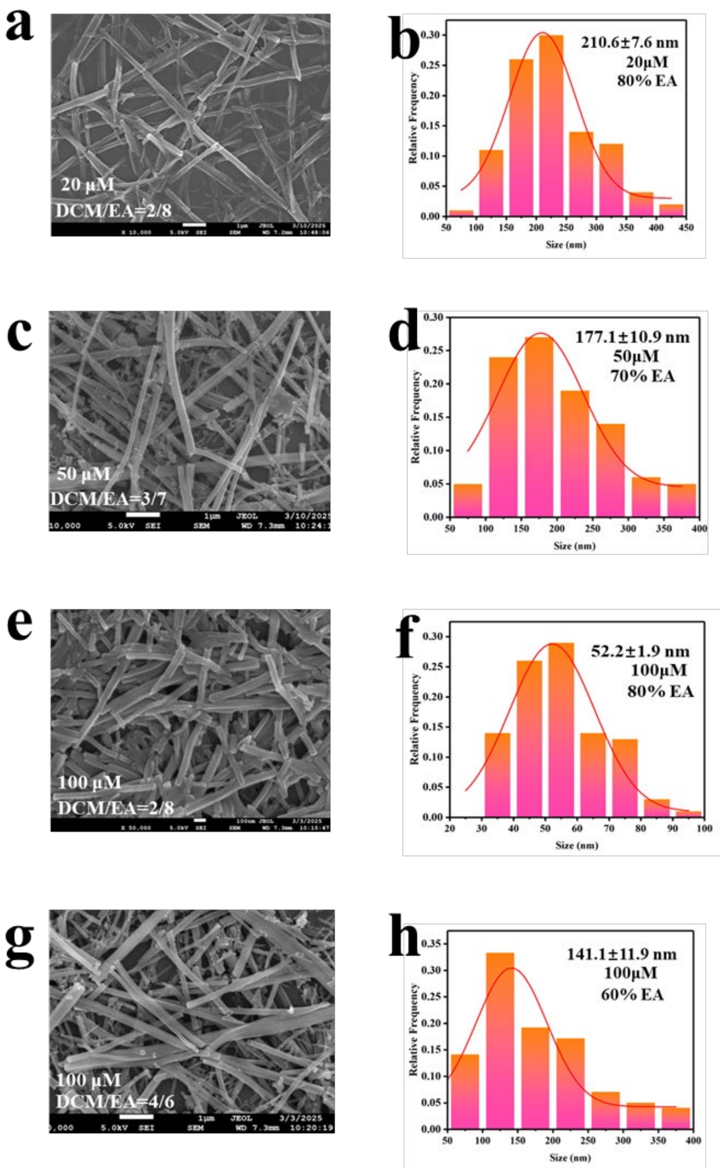

Figure S16. SEM images and corresponding size histogram of MOC 1-based self-assemblies in DCM/EA (a) 20µM, 80%EA, (b) 50µM, 70%EA, (c) 100µM, 80%EA, and (d) 100µM, 60%EA.

3.6 UV–vis and Fluorescence spectra of MOC 2 at different concentrations in DCM

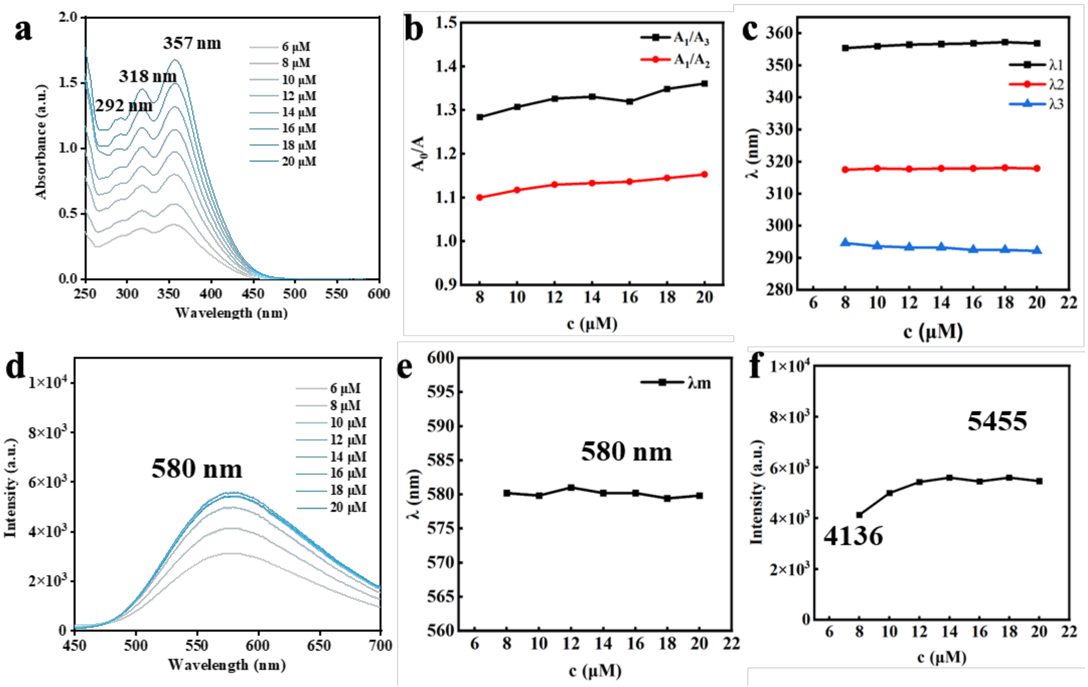

Figure S17. (a) UV–vis spectra of **MOC 2** in DCM (6-20 μM), (b) relationships between **λ** and concentrations, (c) the ratio of A_{357}/A_{287} change with concentration increases. (d) fluorescence spectra of **MOC 2** in DCM (6-20 μM), (e) relationships between λ_{em} and concentrations, (f) the fluorescence intensity with concentration increases.

3.7 SEM images of MOC 2-based self-assemblies

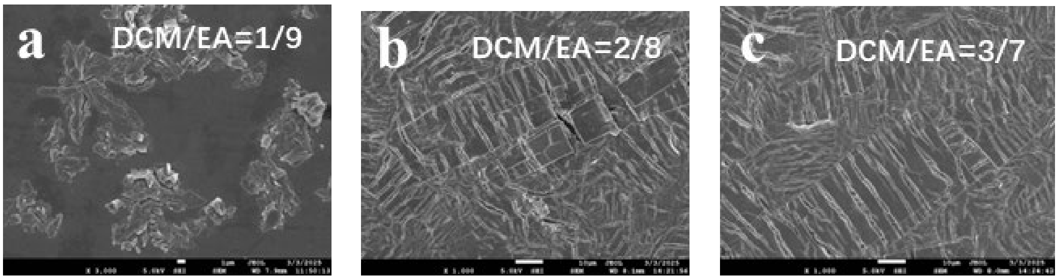

Figure S18. SEM images of **MOC 2**-based self-assemblies in DCM/EA (a) 100μM, 90%EA, (b) 100μM, 80%EA, (c) 100μM, 70%EA.

4. Partial ^{1}H NMR spectrum of MOC 1-2

4.1 Partial variable-temperature ^{1}H NMR spectrum of MOC 1 in CD_2Cl_2

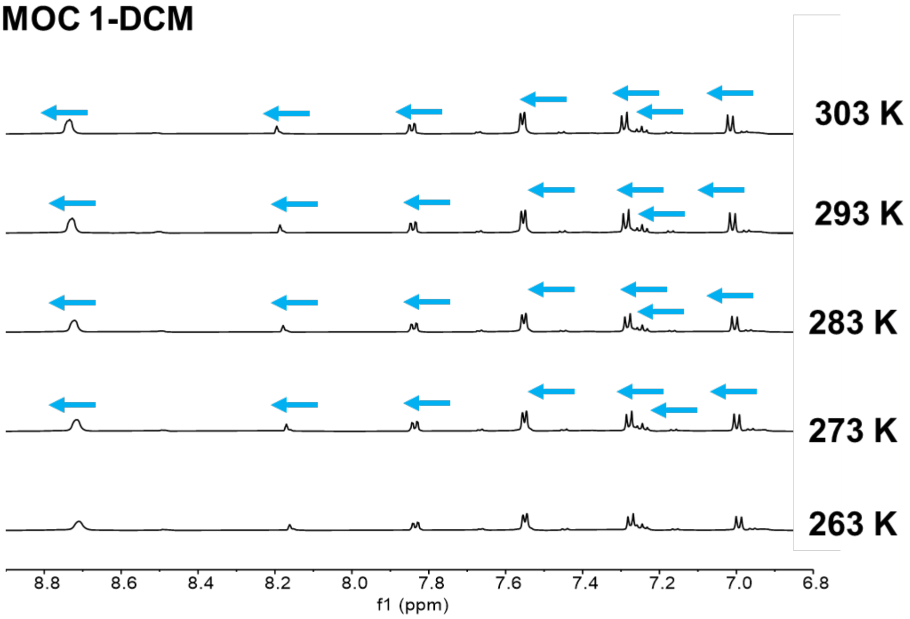

Figure S19. Partial variable-temperature ^{1}H NMR spectrum of **MOC 1** in CD_2Cl_2 from 263K to 303K.

4.2 Partial ^{1}H NMR spectrum of MOC 1 in CD_2Cl_2 and CD_2Cl_2/EA mixtures

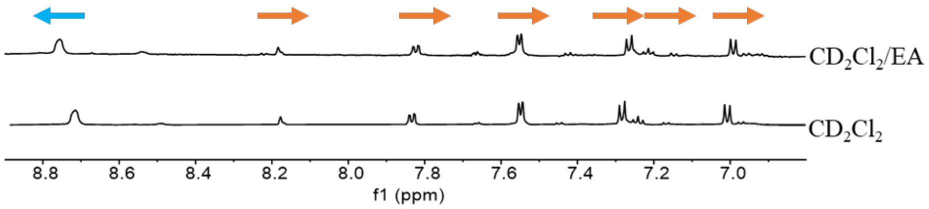

Figure S20. Partial ^{1}H-NMR spectrum of **MOC 1** in CD_2Cl_2 and CD_2Cl_2/EA mixtures at 263K (CD_2Cl_2/EA=9 : 1, 1mM).

4.3 Partial variable-temperature ^{1}H NMR spectrum of MOC 1 in CD_2Cl_2/EA

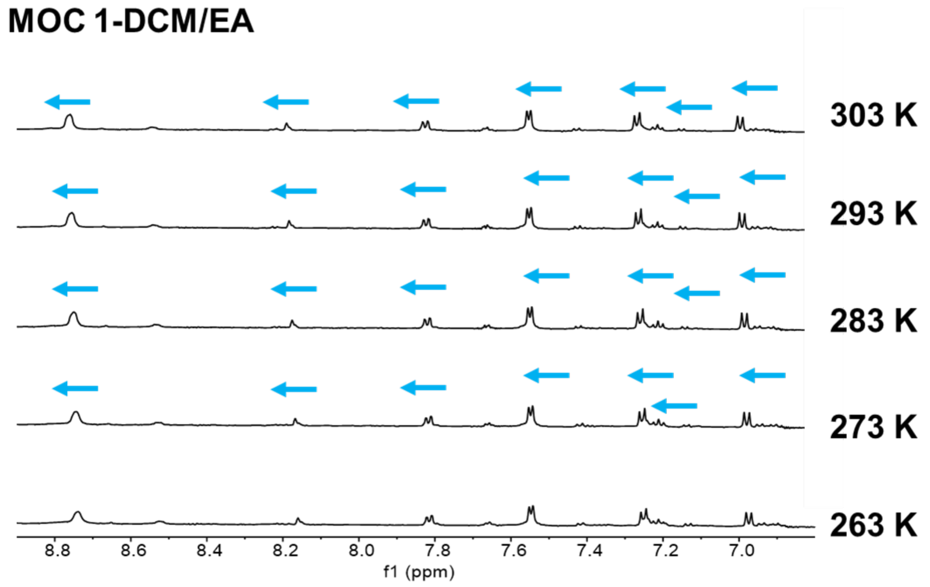

Figure S21. Partial variable-temperature ^{1}H NMR spectrum of **MOC 1** in CD_2Cl_2/EA from 263K to 303K.

4.4 Partial variable-temperature ^{1}H NMR spectrum of MOC 2 in CD_2Cl_2

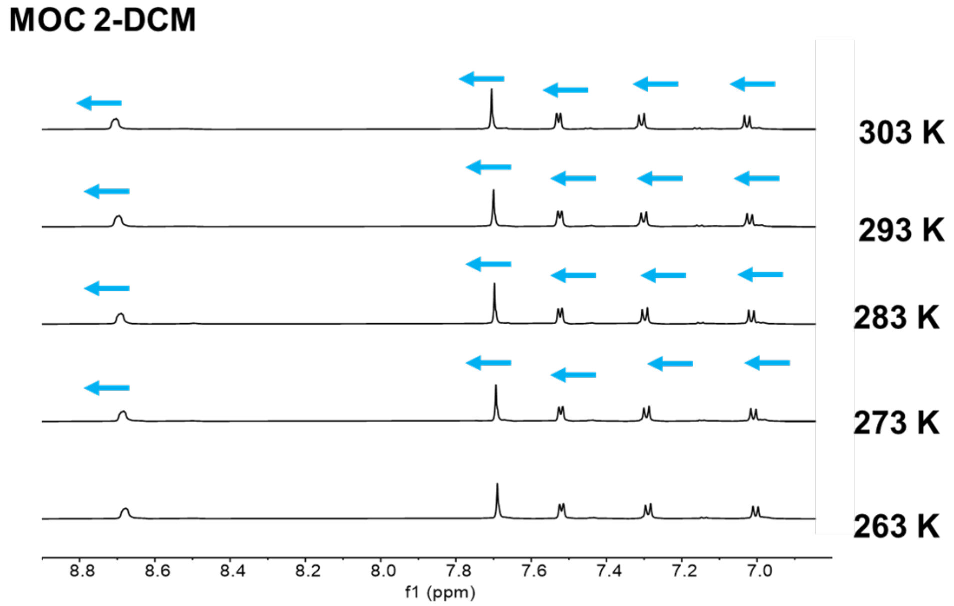

Figure S22. Partial variable-temperature ^{1}H NMR spectrum of **MOC 2** in CD_2Cl_2 from 263K to 303K.

4.5 Partial ^{1}H NMR spectrum of MOC 2 in CD_2Cl_2 and CD_2Cl_2/EA mixtures

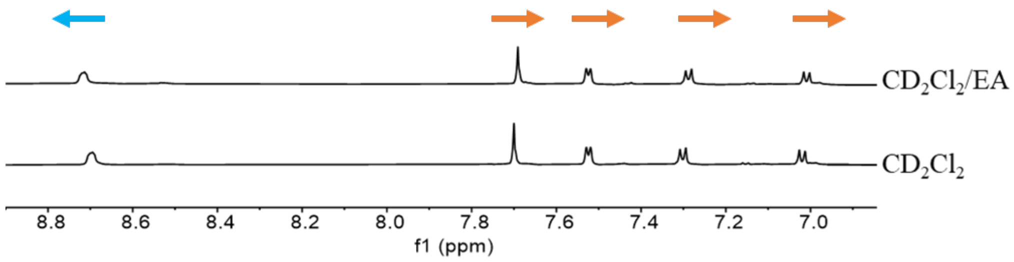

Figure S23. Partial ^{1}H-NMR spectrum of **MOC 2** in CD_2Cl_2 and CD_2Cl_2/EA mixtures at 263K (CD_2Cl_2/EA=9: 1, 1mM).

4.6 Partial variable-temperature ^{1}H NMR spectrum of MOC 2 in CD_2Cl_2/EA

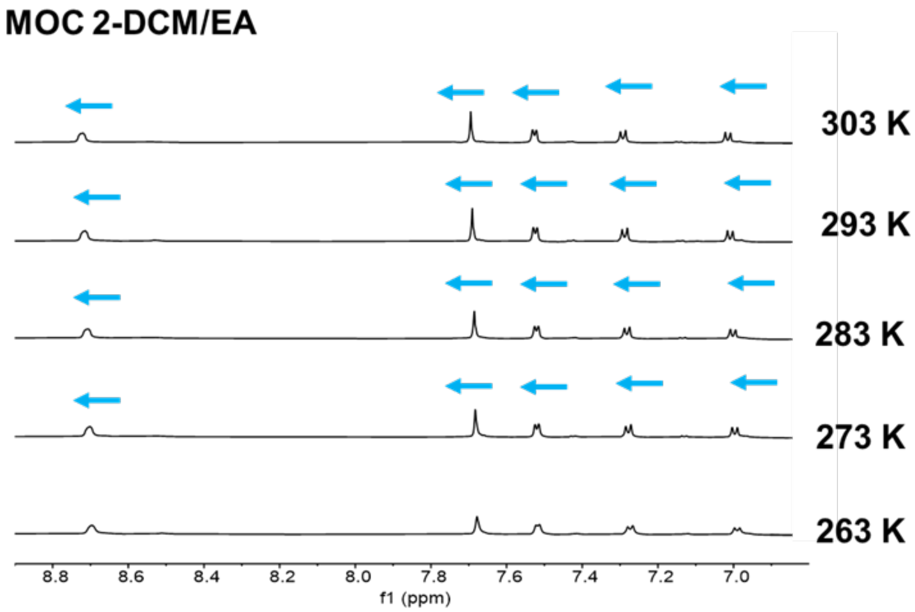

Figure S24. Partial variable-temperature ^{1}H-NMR spectrum of **MOC 2** in CD_2Cl_2/EA from 263K to 303K.

4.7 The XRD pattern of MOC 2 ribbon

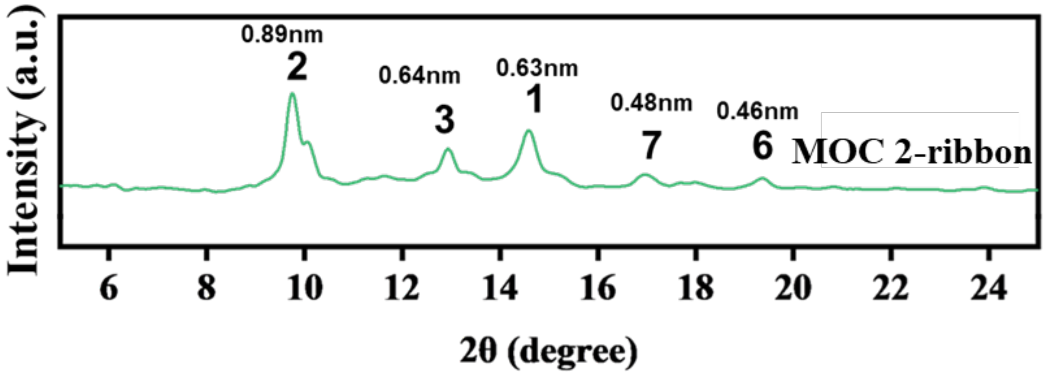

Figure S25. The XRD pattern of **MOC 2** ribbon.

4.8 The XRD pattern of MOC 2 amorphous powders

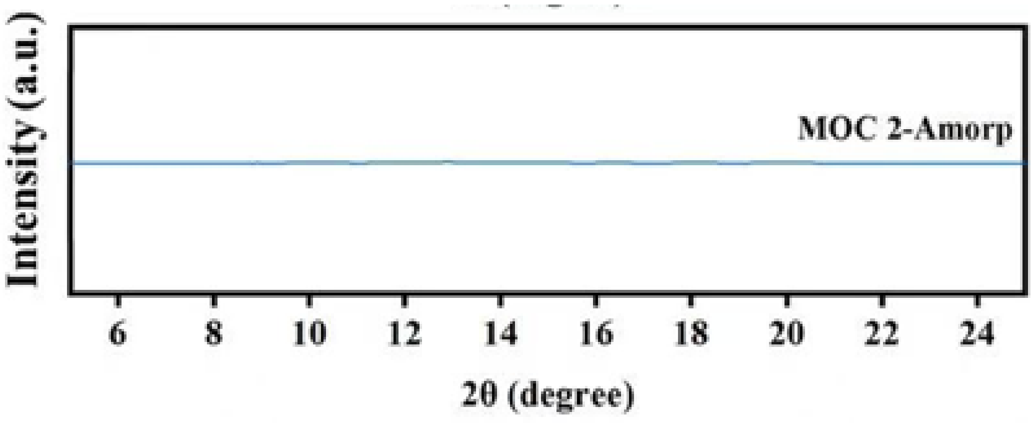

Figure S26. The XRD pattern of **MOC 2** amorphous powders.

4.9 X-ray single-crystal structure of MOC 2

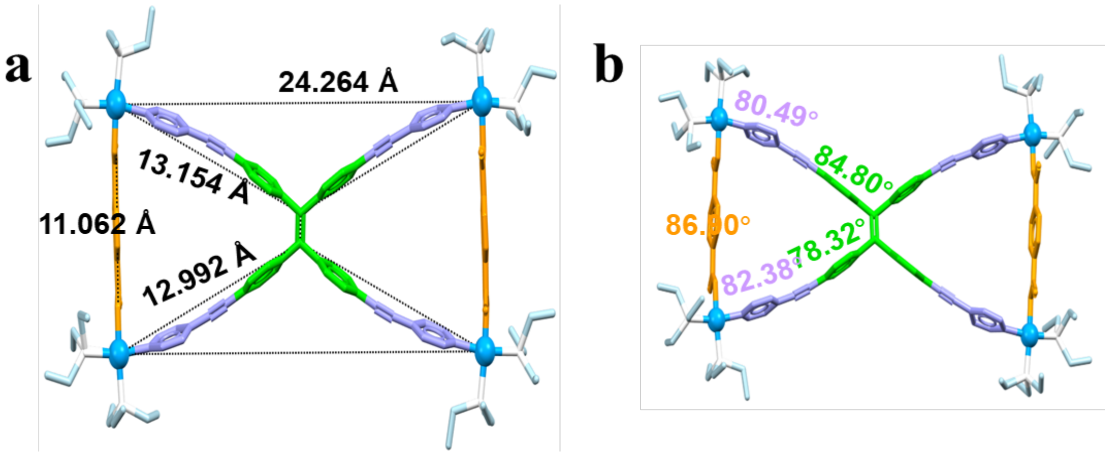

Figure S27. (a) X-ray single-crystal structure of the distances and between benzene rings, pyridine rings, platinum, and phosphorous atoms in **MOC 2**, (b) dihedral angles between benzene rings and pyridine rings in **MOC 2.**

4.10 Stacking mode of MOC 2-based self-assemblies

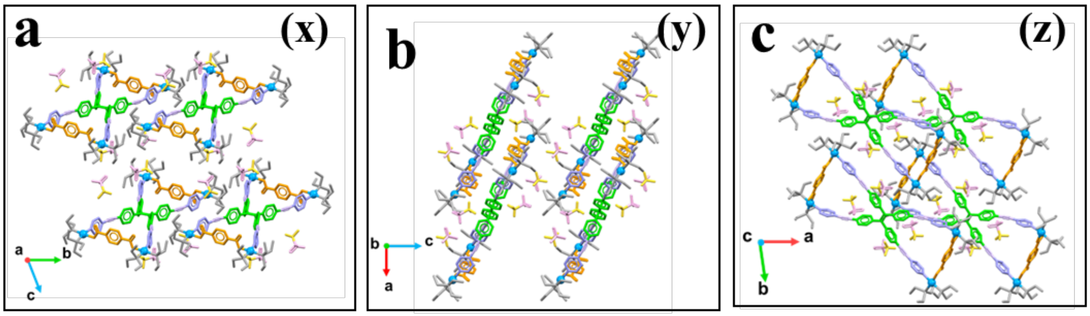

Figure S28. Single crystal of **MOC 2** stacking from a, b, c axis.

4.11 Crystallographic Data for MOC 2

Table 2. Crystallographic Data for **MOC 2**

Compound	MOC 2
Empirical formula	$C_{122}H_{160}F_{12}N_4O_{20}P_8Pt_4S_4$
Formula weight	3386.992
Temperature/K	193
Wavelength	1.34139
Crystal system	triclinic
Space group	P-1
Unit cell dimensions	a=14.066(3) α=68.270(7)°<br>b=17.692(4) β=87.738(8)°<br>c=18.890(4) γ=80.653(7)°
Volume	4307.8(15)
Z	1
Density(calculated)g/cm^3	1.306
Absorption coefficient	5.192
F(000)	1669.8
Crystal size	0.13 × 0.11 × 0.1
Theta range for data collection/°	4.38 to 105.96
Index ranges	-16 ≤ h ≤ 16, -21 ≤ k ≤ 20, -22 ≤ l ≤ 22
Reflections collected	55223
Independent reflections	15023 [Rint = 0.0461, Rsigma = 0.0358]
Completeness to theta	(52.982°)98.7%
Absorption correction	multi-scan
Refinement method	Full-matrix least-squares on F^2
Data /restraints/ parameters	15023/886/1004
Goodness-of-fit on F^2	1.139
Final R indexes [I>2sigma(I)]	R_1 = 0.0648, wR_2 = 0.1922
R indexes (all data)	R_1 = 0.0775, wR_2 = 0.2019
Largest diff. peak and hole	1.82/-1.54

Table S3. Selected hydrogen bond parameters (Å) for **MOC 2**

**D**	**H**	**A**	**d(D-H)/Å**	**d(H-A)/Å**	**d(D-A)/Å**	**D-H-A/°**
C1	H1a	$F5^{1}$	0.98	2.83(13)	3.39(2)	117(10)
C1	H1b	$F1^{2}$	0.98	2.35(11)	3.13(3)	135(11)
C11	H11a	$O7A^{3}$	0.98	2.51(7)	3.10(6)	119(2)
C11	H11a	$O6^{3}$	0.98	2.81(6)	3.305(17)	112(4)
C12	H12a	$O10^{1}$	0.99	2.731(19)	3.334(18)	119.7(4)
C13	H13	$O6^{3}$	0.95	2.454(17)	3.046(17)	120.3(4)
C16	H16	$O1^{4}$	0.95	2.757(11)	3.269(12)	114.6(2)
C17	H17	$O1^{4}$	0.95	2.439(11)	3.110(12)	127.5(2)
C22	H22	$O3^{5}$	0.95	2.426(8)	3.349(8)	163.85(18)
C37	H37	O3	0.95	2.523(11)	3.054(11)	115.4(3)
C39	H39	F2	0.95	2.64(3)	3.16(3)	114.9(4)
C39	H39	O10	0.95	2.455(18)	3.349(18)	156.8(5)
C39	H39	F3A	0.95	2.74(3)	3.33(3)	121.4(5)
C39	H39	O10A	0.95	2.40(2)	3.34(2)	169.1(5)
C55	H55b	O6A	0.99	2.77(6)	3.38(5)	119.8(16)
C58	H58a	O6A	0.99	2.26(8)	2.99(8)	129.1(16)
C58	H58b	$O8A^{6}$	0.99	2.503(19)	3.328(19)	140.7(6)
C48	H48a	O4	0.99	2.07(2)	2.68(2)	118.1(6)
C50	H50a	O4	0.99	2.88(2)	3.37(2)	111.2(4)
C52	H52a	$O5^{7}$	0.99	2.39(3)	3.24(3)	143.4(7)
C53	H53b	$O7^{7}$	0.98	2.44(10)	3.27(3)	143(12)
C57	H57b	O5	0.98	2.38(12)	3.05(3)	125(10)
C48A	H48c	O4	0.99	2.72(2)	3.22(2)	111.8(5)
C50A	H50d	O4	0.99	2.46(2)	3.08(2)	120.2(6)
C52A	H52c	$O8A^{6}$	0.99	2.47(3)	3.35(3)	147.5(7)
C52A	H52d	$O5A^{7}$	0.99	2.00(8)	2.66(7)	121.7(18)
C53A	H53e	$F2A^{6}$	0.98	1.99(12)	2.75(3)	133(12)
C56A	H6Aa	S2A	0.99	2.47(6)	3.25(6)	135.7(13)
C56A	H6Aa	O6A	0.99	1.21(8)	2.10(8)	145(4)
C57A	H57e	S2A	0.98	2.59(5)	3.32(5)	131.0(13)
C57A	H57e	F4A	0.98	1.24(11)	2.10(7)	142(14)

Symmetry transformations used to generate equivalent atoms:

[1]1-X,-Y,1-Z; [2]-1+X,-1+Y,1+Z; [3]2-X,-Y,1-Z; [4]-1+X,-1+Y,+Z; [5]2-X,1-Y,1-Z; [6]1+X,+Y,+Z; [7]3-X,1-Y,-Z

4.12 Intermolecular hydrogen bonds of MOC 2

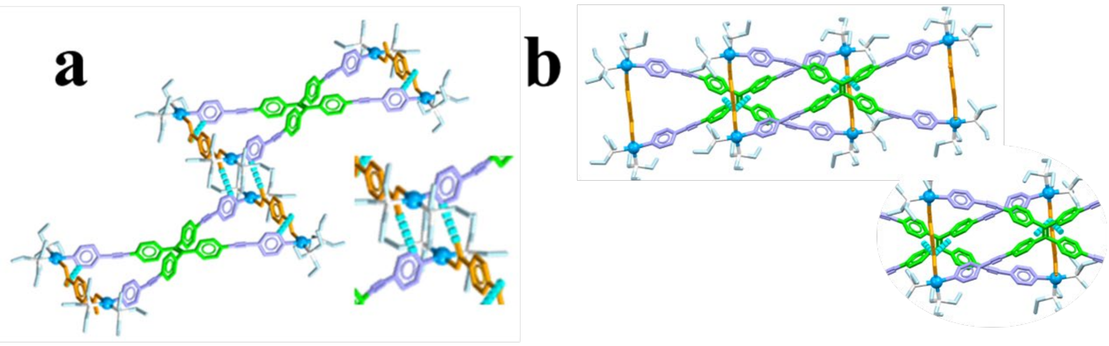

Figure S29. Intermolecular hydrogen bonds of **MOC 2**.

4.13 The binding configurations of ribbon planes and organization of distinct dimer configurations in MOC 2 assemblies

DFT calculations demonstrate that the structural stability of this supramolecular assembly is maintained by two cooperative intermolecular interactions.

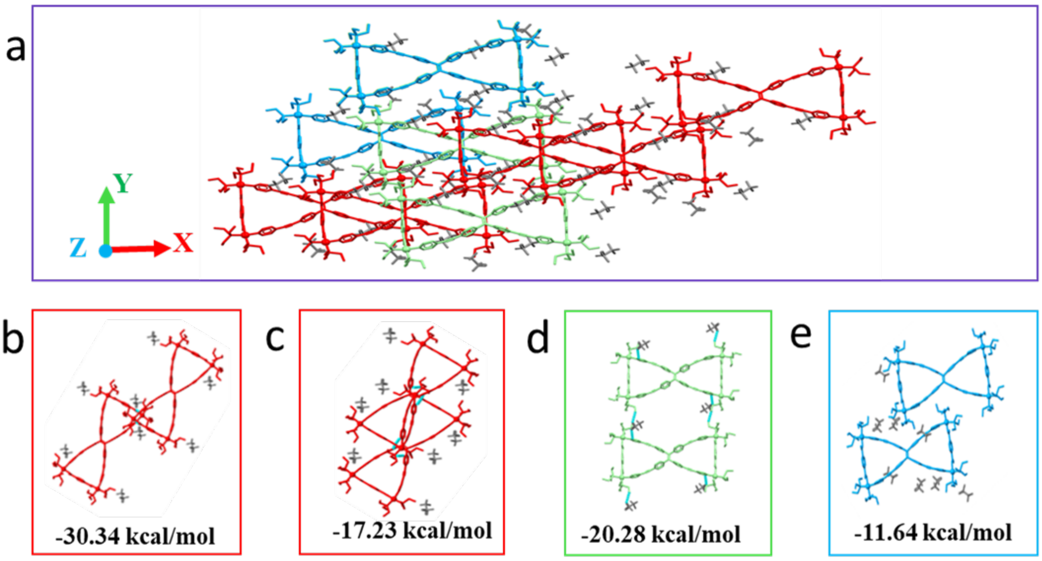

Figure S30. The binding configurations of ribbon planes and organization of distinct dimer configurations in **MOC 2** assemblies.

In the stacking structure of MOC 2, the hydrogen-bond-mediated stacking units 1 (**Figure S30a**) and 2 (**Figure S30c**) exhibit the strongest binding energies (-30.34 and -17.23 kcal/mol, respectively), driving preferential molecular growth along the ribbon extension direction (X-axis). The triflate-mediated stacking 3 (-20.28 kcal/mol) (**Figure S30d**) dominates the extension along the ribbon width direction (Y-axis), while the van

der Waals-interacted stacking 4 (-11.64 kcal/mol) (**Figure S30e**) regulates growth along the thickness direction (Z-axis) perpendicular to the two-dimensional plane. The binding energy differences indicate that the priority of molecular stacking follows the order X > Y > Z, thereby resulting in the formation of a two-dimensional ribbon-like structure.

4.14 Microscopy image of MOC 1-2 assemblies in DCM/Hexane

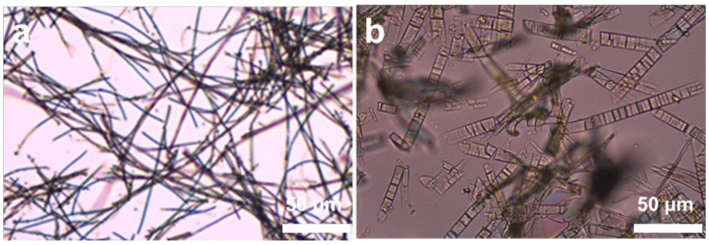

Figure S31. (a) Microscopy image of the fibrous structure self-assembled by MOC 1 in a solution of 100 μM DCM/Hex = 4/6, and (b) microscopy image of the fibrous structure self-assembled by MOC 1 in a solution of 100 μM DCM/Hex = 4/6.

4.15 X-ray single-crystal structure of MOC 1

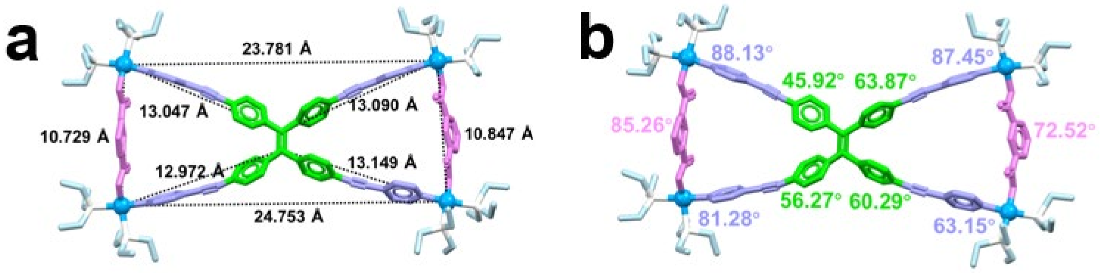

Figure S32. (a) X-ray single-crystal structure of the distances and between benzene rings, pyridine rings, platinum, and phosphorous atoms in **MOC 1**, (b) dihedral angles between benzene rings and pyridine rings in **MOC 1.**

4.16 Stacking mode of MOC 1-based self-assemblies

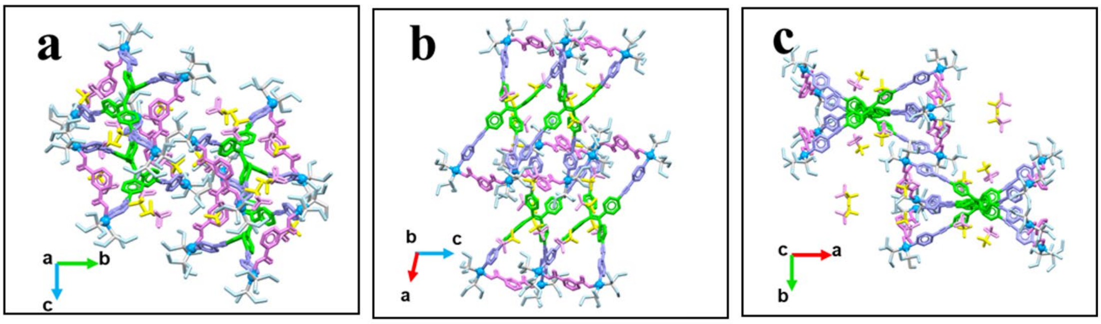

Figure S33. (a-c) Single crystal of **MOC 1** stacking from a, b, c axis.

4.17 Crystallographic Data for MOC 1

Table S4. Crystallographic Data for MOC 1

Compound	MOC 1
Empirical formula	$C_{122}H_{160}F_{12}N_4O_{20}P_8Pt_4S_4$
Formula weight	3386.992
Temperature/K	193
Wavelength	1.54178
Crystal system	monoclinic
Space group	$P2_1/c$
Unit cell dimensions	a=33.396(3) α=90°<br>b=30.230(4) β=102.090(5)°<br>c=18.7796(13) γ=90°
Volume	18539(2)
Z	4
Density(calculated)g/cm^3	1.213
Absorption coefficient	7.085
F(000)	6736.0
Crystal size	0.12 × 0.1 × 0.08
Theta range for data collection/°	3.982 to 108.474
Index ranges	$-35 \leq h \leq 35$, $-31 \leq k \leq 27$, $-19 \leq l \leq 19$
Reflections collected	80584
Independent reflections	22540 [Rint = 0.1763, Rsigma = 0.1290]
Completeness to theta	(108.47°)99.5%
Absorption correction	multi-scan
Refinement method	Full-matrix least-squares on F^2
Data /restraints/ parameters	22540/2326/1677
Goodness-of-fit on F^2	1.084
Final R indexes [I>2sigma(I)]	$R_1 = 0.1070$, $wR_2 = 0.2887$
R indexes (all data)	$R_1 = 0.1616$, $wR_2 = 0.3327$
Largest diff. peak and hole	3.37/-1.60

Table S5. Selected hydrogen bond parameters (Å) for MOC 1

D	H	A	d(D-H)/Å	d(H-A)/Å	d(D-A)/Å	D-H-A/°
C1	H1	O1[1]	0.95	2.50(3)	3.06(3)	117.8(6)
C2	H2	O1[1]	0.95	2.40(3)	3.01(3)	121.9(5)
C5	H5	F1_11	0.95	2.60(4)	3.27(3)	128.2(8)
C5	H5	O1_11	0.95	2.57(3)	3.23(3)	126.7(7)
C28	H28	O1_10	0.95	2.60(2)	3.19(2)	120.4(6)
C36	H36	O5[2]	0.95	2.72(2)	3.22(2)	114.1(5)
C37	H37	O5[2]	0.95	2.53(2)	3.14(2)	122.2(5)
C39	H39	O1_9	0.95	2.43(3)	3.18(3)	136.0(7)
C45	H45	O2_12	0.95	2.72(4)	3.25(4)	116.3(7)
C5_1	H5a_1	O2_10[3]	0.99	2.52(2)	3.12(2)	118.6(6)
C6_1	H6a_1	O1_9	0.98	2.36(2)	3.25(2)	150.8(6)
C3_2	H3a_2	O2_10[3]	0.99	2.31(2)	3.21(2)	150.8(6)
C3_4	H3a_4	O8	0.99	2.64(2)	3.14(2)	111.5(4)
C5_4	H5a_4	O8	0.99	2.596(19)	3.19(2)	118.3(4)
C6_4	H6b_4	F3_9[4]	0.98	2.59(3)	3.10(3)	113.0(6)
C4_7	H4b_7	F2_12[5]	0.98	2.36(6)	3.16(6)	138.3(12)
C6_13	H6b_13	S1_11[6]	0.98	2.18(10)	3.09(9)	155(2)
C6_13	H6b_13	O1_11[6]	0.98	2.34(10)	3.24(9)	151.5(10)
C6_13	H6b_13	O2_11[6]	0.98	1.58(6)	2.37(6)	135(3)

Symmetry transformations used to generate equivalent atoms:
[1]-X, -Y,1-Z; [2]1-X,1-Y,1-Z; [3]1-X,1/2+Y,3/2-Z; [4]+X,1/2-Y,-1/2+Z; [5]-X,-1/2+Y,3/2-Z; [6]+X,+Y,1+Z

4.18 The XRD pattern of MOC 1 fibers

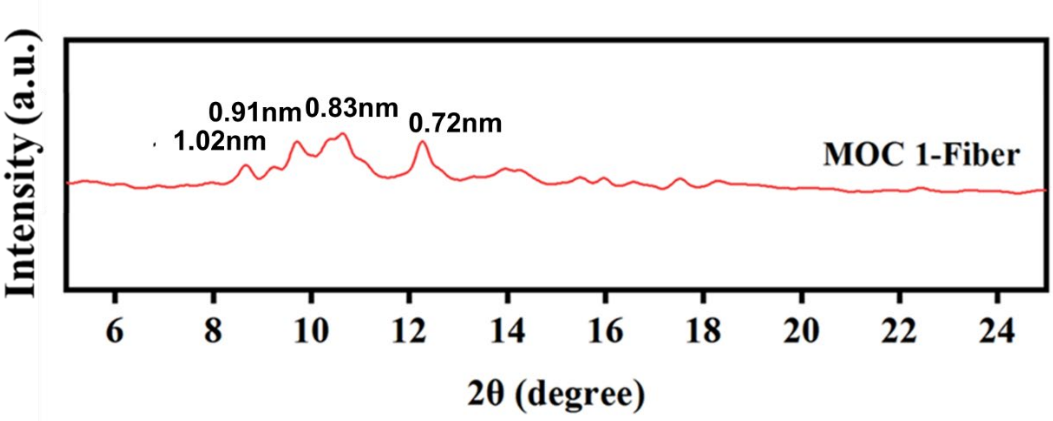

Figure S34. The XRD profile of **MOC 1** fibers.

4.19 The XRD pattern of MOC 1 amorphous powders

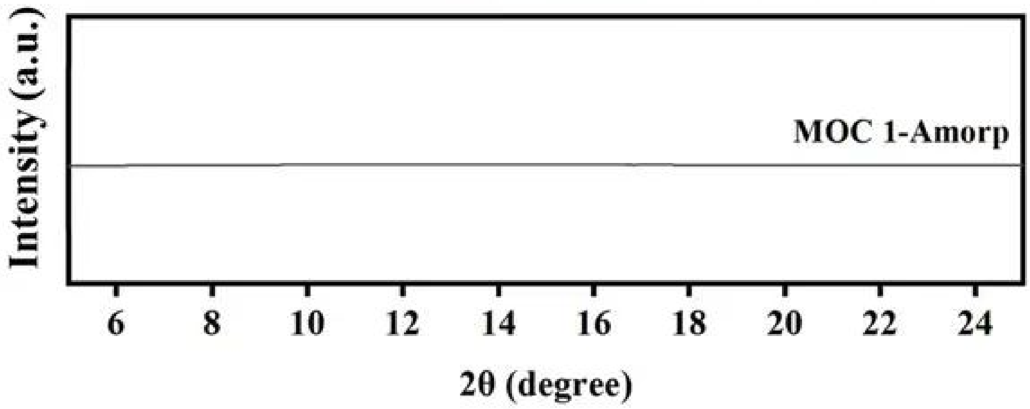

Figure S35. The XRD profile of **MOC 1** amorphous powders.

4.20 Interaction energies and energy decomposition for MOC 2

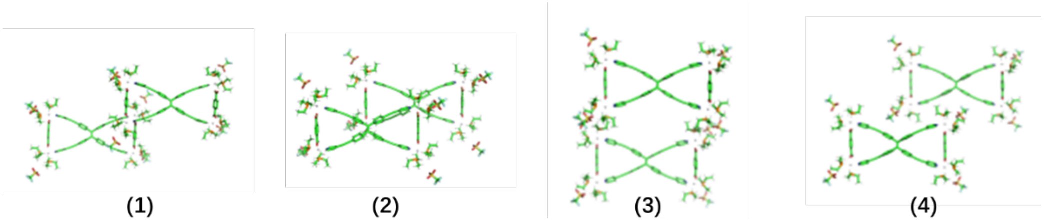

Figure S36. Organization of 4 distinct dimer configurations.

All calculations were performed using Gaussian 16 software[8], with the b3lyp level of theory[9]. When calculating the interaction between dimers, we used the crystal configuration directly to perform single-point energy calculations. The calculations utilized a mixed basis set, where N, C, O, H, S, P, and F atoms employed the 6-31+g(d,p) basis set, and Pt used the Lanl2dz pseudopotential basis set. Energy decomposition was performed using the sobEDA program[10]. During the single point calculation, the SCF energy convergence criterion is set as 10^{-6} a.u. All the structure of molecules were plotted using PyMOL software[11].

Table S6. Interaction energies and energy decomposition for the dimer configurations.

Dimer index	Energy (kcal/mol)				
	Total	Electronic	Exchange-repulsion	Orbital	Dispersion
1	-30.34	-12.76	21.67	-6.11	-33.14
2	-17.23	8.33	29.27	-7.57	-47.27
3	-20.28	-4.87	15.87	-8.63	-22.65
4	-11.64	-7.7	0.89	-1.46	-3.37

4.21 Fluorescence lifetime images of MOC 1-2 Assemblies in DCM/Hexane

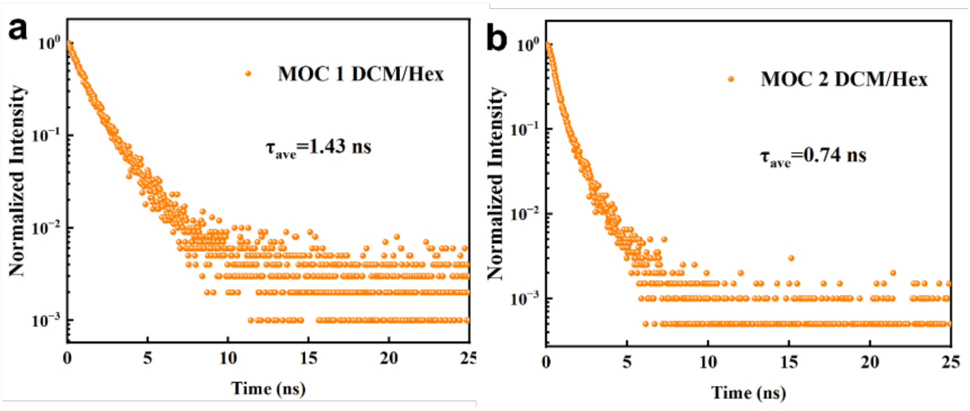

Figure S37. (a) Fluorescence lifetime image of the MOC 1 fibrous superstructure assembled in the DCM/Hex solution, and (b) fluorescence lifetime image of the MOC 2 ribbon-like superstructure assembled in the DCM/Hex solution.

References

[1] J. G. Brandenburg, C. Bannwarth, A. Hansen, S. Grimme, S. Ehrlich, *J. Phys. Chem. A*, 2018, **148**, 064104.

[2] J.-D. Chai and M. Head-Gordon, *J. Chem. Phys.*, 2008, **128**, 084106.

[3] S. Grimme, J. Antony, S. Ehrlich and H. Krieg, *J. Chem. Phys.*, 2010, **132**, 154104.

[4] F. Weigend and R. Ahlrichs, *Phys. Chem. Chem. Phys.*, **2005**, 7, 3297–3305.

[5] F. Neese, *Wiley Interdiscip. Rev.: Comput. Mol. Sci.*, 2022, **12**, e1606.

[6] T. Lu and F. Chen, *J. Comput. Chem.*, 2012, **33**, 580–592.

[7] T. Lu, *The J. Chem. Phys.*, 2024, **161**, 082503.

[8] Frisch, M.J. et al. Gaussian 16 Rev. C.01. Gaussian, Inc., 2016.

[9] R. Bauernschmitt, and R. Ahlrichs, *Chem. Phys. Lett.*, 1996. **256**, 454-464.

[10] T. Lu and Q. Chen, *J. Phys. Chem. A*, 2023. **127**, 7023-7035.

[11] L. Schrödinger and W. DeLano, PyMOL. 2020.
